# Plasma small-extracellular vesicles’ proteomic signature in neoadjuvant chemotherapy–naïve breast cancer patients

**DOI:** 10.1371/journal.pone.0348500

**Published:** 2026-05-05

**Authors:** Amr Ahmed WalyEldeen, Ghada Mohamed, Sherif Nasser Taha, Abdallah M. Gameel, Liali Yousef Talat, Hebatallah Hassan, Sherif Abdelaziz Ibrahim

**Affiliations:** 1 Department of Zoology, Faculty of Science, Cairo University, Giza, Egypt; 2 Department of Pathology, National Cancer Institute, Cairo University, Cairo, Egypt; 3 Department of Surgical Oncology, National Cancer Institute, Cairo University, Cairo, Egypt; 4 Department of Surgical Oncology, Baheya Centre for Early Detection and Treatment of Breast Cancer, Giza, Egypt; 5 Clinical Pathology Department, National Cancer Institute, Cairo University, Cairo, Egypt; Qatar Biomedical Research Institute, QATAR

## Abstract

Breast cancer remains a leading cause of cancer-related mortality worldwide, with obesity markedly increasing the risk in affected individuals. Liquid biopsy-based extracellular vesicles (EVs) offer a minimally invasive platform for molecular profiling of tumor-derived markers. Plasma small-EVs from obese, chemotherapy-naïve breast cancer patients (n = 76; stages I–III) and age-matched obese controls (n = 36) were enriched and characterized by high-resolution transmission electron microscopy, dynamic light scattering (DLS) and specific EV markers. Proteomic profile of the enriched small-EVs using nanoLC-MS/MS identified Fibronectin 1 (FN1) and von Willebrand Factor (VWF) as candidate markers. Bioinformatics and STRING networks revealed interactions with Syndecan-2 (SDC2) and Galectin-3 (Gal-3). As an independent validation, western blot confirmed that FN1, VWF, and SDC2 were higher enriched in the small-EVs of breast cancer with different stages than in those of normal and that high content of small-EVs FN1 and SDC2 was primarily associated with the aggressive triple-negative breast cancer (TNBC) subtype. Interestingly, Gal-3 was reduced in small-EVs but elevated in breast carcinoma tissues and microvesicle (MV)-enriched EVs. Functionally, treatment with TNBC-derived plasma small-EVs not only downregulated expression of epithelial marker *CDH1, and* upregulated expression of the mesenchymal markers *ZEB2* and FN1 in low-invasive MCF-7 breast cancer cells, but also elevated expression of inflammatory and matrix-remodeling mediators (*Il-6*, *Tnf-α*, and *Mmp-9*) in BNL CL.2 normal liver cells. ROC-Plotter and drug–gene interaction analyses indicated associations with therapy response, with approved compounds targeting FN1 and VWF. Overall, these findings reveal proteomic signatures of minimally invasive plasma small-EVs as promising markers associated with diagnosis, molecular subtyping, disease progression, and guiding therapeutic strategies in obese breast cancer patients.

## 1. Introduction

Breast cancer remains one of the most prevalent malignancies worldwide, representing a major cause of morbidity and mortality among women [1]. Breast cancer is commonly classified using immunohistochemical surrogate markers into four major subtypes: luminal A (LumA; estrogen receptor [ER]-positive, progesterone receptor [PR]-positive, human epidermal growth factor receptor 2 [HER2]-negative, with a low Ki-67 index), luminal B (LumB; ER-positive, PR-positive, HER2-negative or HER2-positive, with a high Ki-67 index), HER2-enriched (ER-negative, PR-negative, and HER2-overexpressing), and basal-like/triple-negative breast cancer (TNBC; ER-negative, PR-negative, and HER2-negative) [2].

Despite substantial progress in early detection, targeted therapies, and improved survival rates, breast cancer continues to pose significant clinical challenges especially for patients with advanced or metastasis [3]. These challenges are further complicated by obesity, a major risk factor that contributes to cancer progression and treatment resistance through chronic inflammation, hormonal imbalance, and altered metabolic states [4]. Obesity has been shown to drive extracellular matrix (ECM) remodeling, with increased deposition of basement membrane components, collagen crosslinkers, and angiogenic factors, all of which create a microenvironment conducive to tumor invasion and metastasis [5]. Egypt ranks among the countries with the highest obesity burden worldwide, placing 18th globally in obesity prevalence [6]. Adult obesity in Egypt rose to approximately 40% according to the 100 million Health Survey in 2019, up from an estimated 36% in 2017 [6]. Also, obesity is correlated with lymph node status, larger tumors, metastasis, poorer survival rates, and reduced disease-free survival [7]. Additionally, obesity increases the likelihood of developing a secondary tumor in previously diagnosed breast cancer patients, especially within the opposite breast, endometrium, and colon [8]. Taken together, these findings highlight a critical unmet need for innovative, non-invasive diagnostic tools to improve the precision and effectiveness of breast cancer management.

Liquid biopsy offers a non-invasive approach in cancer diagnostics to detect molecular changes associated with tumor progression [9]. Among the various analytes used in liquid biopsy, extracellular vesicles (EVs), particularly small-EVs, have gained considerable attention because they carry biomolecules including proteins, lipids, and nucleic acids [10]. Small-EVs are membrane-bound vesicles typically measuring <200 nm in diameter and are secreted by most cell types into biological fluids including blood, urine, and saliva [11]. In cancer biology, small-EVs facilitate intercellular communication and play pivotal roles in tumor growth, metastasis, immune evasion, and drug resistance [12]. Small-EVs play a crucial role in delivering molecular cargo involved in the establishment of pre-metastatic niches (PMNs) at organ-specific sites, where these niches provide a favorable environment for tumor cell colonization [13]. Unlike circulating free proteins, small EV proteins are encapsulated within vesicles, which protect them from degradation and may facilitate their reliable detection in biofluids [14].

In this study, we performed a comprehensive proteomic profiling of plasma-derived small-EVs isolated from obese, chemotherapy-naïve breast cancer patients across clinical stages I-III and molecular subtypes. Small-EVs were isolated using a polyethylene glycol (PEG)-based precipitation method, and their protein content was characterized through different experimental and bioinformatics approaches. Ultimately, we aimed to uncover novel small-EVs biomarkers with potential utility for early detection and treatment stratification, particularly in high-risk populations, such as obese breast cancer patients.

## 2. Materials and methods

### 2.1. Human blood samples

Adult obese female patients (BMI > 30) with confirmed primary breast cancer (stages I-III, non-metastatic) were recruited for this study. All included patients underwent either a modified radical mastectomy or breast conservative surgery and are neoadjuvant chemotherapy naive. Exclusion criteria included patients with metastatic disease at diagnosis (stage IV), significant comorbid conditions such as autoimmune diseases, infections (HIV, HCV, and HBV), and hemolyzed samples. A total of 76 eligible breast cancer patients were included in the study. The study also included 36 age-matched normal obese controls. All control participants were initially subjected to a cancer screening process involving a clinical examination. In cases where suspected detectable breast lumps or a notable familial history of breast cancer, mammographic evaluation was conducted for further assessment. Only individuals with no evidence of breast malignancy or clinically relevant benign breast disease were considered eligible for inclusion in the control group. Patient and control recruitment was conducted between 26 April 2022 and 25 April 2023 upon obtaining approval for this study from the Baheya Research Ethics Committee’s institutional review board (IRB protocol number: 202204260017) at the Baheya Centre for Early Detection and Treatment of Breast Cancer, Giza, Egypt. All participants provided written informed consent to participate in the study, and all methods were carried out in accordance with relevant regulations and with the Declaration of Helsinki. Clinicopathological data, including patient age, BMI, family history, tumor laterality, size, grade, lymph node status, stage, and molecular subtypes, were collected from their medical records. HER2 status was assessed using silver in situ hybridization (SISH) following established procedures [15]. Estrogen receptor (ER), progesterone receptor (PR), HER2 immunohistochemistry (IHC), and SISH evaluations were interpreted according to the updated American Society of Clinical Oncology/College of American Pathologists (ASCO/CAP) 2018 guidelines [16]. HER2 amplification detected by SISH was considered HER2-positive, whereas non-amplified cases were classified as HER2-negative.

### 2.2. Enrichment of small-EVs

Peripheral blood samples were obtained in Acid Citrate Dextrose (ACD) tubes to prevent coagulation and reduce platelet activation prior to the patients undergoing curative surgery. Plasma was used for extracellular vesicle isolation instead of serum because clot formation during serum preparation can activate platelets and release additional platelet-derived vesicles, altering the native circulating EV profile and reducing the reliability of downstream analyses [17]. Platelet-poor plasma was obtained by subjecting samples to two sequential centrifugation steps at 2,500 × g for 15 min each. To reduce contamination from larger vesicles, the plasma was subsequently centrifuged at 21,000 × g for 1 h and then mixed at a 1:1 ratio with 0.22 μm-filtered PBS. After passing through a 0.22 μm filter, isolation of small-EVs was carried out using an adapted PEG-based precipitation protocol [18]. In brief, the diluted plasma was incubated overnight at 4 °C with an equal volume of 16% PEG-6000, after which the mixture was centrifuged at 10,000 × g for 10 min. The collected small-EV pellet was then washed with PBS and subjected to a second overnight incubation at 4 °C in PEG solution, followed by a final centrifugation at 10,000 × g for 10 min. The small EV pellet was either resuspended in 200 µL PBS or lysed in 1 × RIPA buffer containing a protease inhibitor cocktail. The protein concentration of the small-EVs preparations was determined using the Bradford assay.

### 2.3. Characterization of the enriched small-EVs

#### 2.3.1. High-resolution transmission electron microscopy.

High-resolution transmission electron microscopy (HR-TEM) analysis was performed at the electron microscopy core facility of the National Research Center, Dokki, Giza, Egypt. A small volume of the resuspended small-EVs was placed on a carbon-coated copper grid and allowed to air dry. Afterward, a few drops of 1% phosphotungstic acid were added to the grid and allowed to dry. The prepared grid was then analyzed using a JEOL JEM-2100 HR-TEM (Tokyo, Japan), operating at a voltage of 200 kV.

#### 2.3.2. Dynamic light scattering (DLS).

DLS was performed at the Faculty of Agriculture core facility, Cairo University, Giza, Egypt. Small-EVs samples were mixed well. Particle size and polydispersity index (PDI) were determined using a Zetasizer instrument (ZEN 3600, Malvern, Worcestershire, UK), The instrument was operated at 25 °C with a measurement duration of 60 s, a count rate of 305.8 kcps, and a measurement position of 4.65 mm.

### 2.4. Western Blot

Protein samples (30–50 µg) were resolved on a 7.5–15% SDS-PAGE gel and subsequently transferred onto a nitrocellulose membrane using a semi-dry electrotransfer technique [19]. Membranes were blocked for 1 hour at room temperature using a 5% skimmed milk (Serva, Heidelberg, Germany) in TBST buffer (200 mM Tris, 150 mM NaCl, 0.1% Tween 20) and rinsed thrice with TBST, for 5 min each. All antibodies used in this study were applied at a working dilution of 1:1000 unless otherwise specified. Primary antibodies against CD9, fibronectin (FN1), von Willibrand factor (VWF) syndecan-1 (SDC1), SDC2, SDC4, protein S (PROS1), galectin-3 (Gal-3), and β-Actin, as listed in S1 Table, were incubated overnight at 4 °C. After washing, membranes were incubated for 1 h at room temperature with the appropriate horseradish peroxidase (HRP)-conjugated secondary antibody, as listed in S1 Table. The protein bands were visualized and imaged by enhanced chemiluminescence (ECL) HRP substrate (Thermo Scientific, Waltham, USA) and the UVP Biospectrum Imaging System (Analytik Jena, Cambridge, UK). CD9, as a loading control for small-EVs was used. Because CD9 detection required non-reducing conditions, the same small EV lysate was run separately for CD9 immunoblotting under non-reducing conditions. A pooled total cell lysate of human breast cancer cell lines was used as positive control, and BSA was used as a negative control, as described before [20].

### 2.5. Dot Blot

Dot blot analysis was carried out to assess small-EV marker proteins. Equal protein content in a volume of 2 µL were spotted onto a nitrocellulose membrane (Amersham, UK) and left to dry at room conditions. The membranes were then subjected to blocking for 1 h at room temperature with 5% (w/v) skimmed milk (Serva, Heidelberg, Germany) dissolved in TBST buffer (10 mM Tris, 150 mM NaCl, 0.1% Tween-20). Following the blocking step, the membranes were washed three times with TBST, for 5 min per wash. The membranes were subsequently incubated at 4 °C overnight with the relevant primary antibodies, including ALIX, HSP70, and CALNEXIN, as listed in S1 Table. All remaining procedures were then completed according to the western blot protocol described previously.

### 2.6. Proteomics analysis

#### 2.6.1. Sample preparation, protein digestion, and peptide cleanup.

Discovery proteomic profiling was performed on a screening cohort (n = 25), comprising stage I (n = 5), stage II (n = 10), and stage III (n = 10) cases. Proteomic profiling of plasma-derived small-EVs lysates was performed at the Proteomics and Metabolomics Research Program, Children’s Cancer Hospital Egypt 57357 (Cairo, Egypt). Small-EVs protein samples in 8 M urea (500 mM Tris-HCl, pH 8.5) were vortexed thoroughly, followed by centrifugation at 10,000 rpm for 30 min to remove insoluble debris. Protein concentration was quantified using the bicinchoninic acid (BCA) assay according to the manufacturer’s instructions.

For in-solution digestion, proteins were reduced with 200 mM dithiothreitol (DTT) for 45 min at room temperature, followed by alkylation with 1 M iodoacetamide (IAA) for 45 min in the dark. Samples were subsequently diluted with 100 mM Tris-HCl (pH 8.5) to reduce urea concentration prior to enzymatic digestion. Proteins were digested overnight at 37 °C with modified porcine trypsin (with ratio 1:30) under continuous shaking. Digestion was terminated by acidification with 100% formic acid to a final pH of 2–3, followed by centrifugation to remove particulates.

Peptide desalting and cleanup were performed using reversed-phase StageTips (MonoSpin C18 columns). Columns were activated with methanol, equilibrated with 0.2% formic acid (solution A), and peptides were loaded, washed, and eluted using 0.2% formic acid in 80% acetonitrile (solution B). Eluted peptides were dried using a vacuum concentrator and reconstituted in solution A prior to LC–MS/MS analysis. Peptide concentration was reassessed using BCA assay to ensure equal injection amounts.

#### 2.6.2. Nano-LC–MS/MS analysis.

Peptide separation and mass spectrometric analysis were performed using an Eksigent nanoLC 400 autosampler coupled to an Ekspert nanoLC 425 pump and interfaced with a Sciex TripleTOF™ 5600 + mass spectrometer. Approximately 1 µg of peptide per sample was injected in trap-and-elute mode onto a CHROMXP C18CL trapping cartridge (5 µm, 10 × 0.5 mm), followed by separation on a CHROMXP C18 analytical column (3 µm, 120 Å, 150 × 0.3 mm).

Peptides were eluted over a 55-minute linear gradient using mobile phase A (0.1% formic acid in water) and mobile phase B (0.1% formic acid in acetonitrile) at a flow rate of 5 µL/min. The mass spectrometer was operated in positive ion mode using data-dependent acquisition (DDA). A high-resolution TOF-MS survey scan (m/z 400–1250) was followed by MS/MS fragmentation (m/z 170–1500) of the 40 most intense precursor ions per cycle, with a total cycle time of 1.5 s. External calibration was performed using Sciex tuning solution to ensure mass accuracy and instrument stability throughout the runs.

Raw MS files were acquired using Analyst TF software (version 1.7.1) and processed using ProteinPilot software (version 5.0.1) employing the Paragon algorithm. Database searching was conducted against the UniProt Homo sapiens reference proteome (Swiss-Prot and TrEMBL). Search parameters included trypsin as the digestion enzyme, iodoacetamide as a fixed cysteine modification, and biological modifications enabled. Bias correction was applied during analysis.

To ensure high-confidence protein identification, false discovery rate (FDR) analysis was enabled, and results were filtered at a maximum FDR of 1% at the protein level using an integrated decoy-based approach. Only proteins meeting the FDR threshold were retained for downstream analyses. Technical quality control included monitoring total ion chromatograms, mass accuracy, and reproducibility of peptide identifications across runs.

### 2.7. Microvesicle-enriched EV enrichment

Microvesicle (MV)-enriched EV fraction used in this study has been enriched and characterized in our recent published study [19]. To further validate MV enrichment, western blotting was performed to assess CD9 marker.

### 2.8. Cell culture

MCF-7 cells were kindly provided by Prof. Dr. Martin Götte (Department of Gynecology and Obstetrics, Münster University Hospital, Münster, Germany), whereas BNL CL.2 mouse normal liver cells were purchased from Nawah Scientific (Cairo, Egypt). Both cell lines were maintained under sterile conditions in a humidified incubator at 37 °C with 5% CO₂. MCF-7 cells were cultured in RPMI-1640 and BNL CL.2 cells in DMEM, each supplemented with 10% fetal bovine serum (FBS) and 1% penicillin–streptomycin (100 U/mL penicillin and 100 µg/mL streptomycin). The culture medium was changed every 2–3 days, and cells were subcultured at approximately 70–80% confluence using 1X trypsin/EDTA. For all experiments, cells were seeded at the indicated densities and allowed to adhere overnight before any treatment. Cell viability was routinely assessed using Trypan blue exclusion (Cat. No. 15250061; Gibco). All cell lines were regularly tested for mycoplasma negative.

### 2.9. Treatment of MCF-7 and BNL CL.2 cells with normal and TNBC plasma-derived small-EVs

MCF-7 and BNL CL.2 cells were seeded in 6-well plates at 4 x 10^5^/well and allowed to adhere overnight to reach ~70–80% confluence. Prior to small-EVs exposure, cultures were gently washed twice with sterile PBS and maintained in their respective serum-free basal media (RPMI-1640 for MCF-7; DMEM for BNL CL.2) to minimize interference from FBS-derived vesicles [21]. Cells were then treated with plasma-derived small-EVs isolated from TNBC patients (TNBC-small-EVs) or normal controls (N-small-EVs) at a final concentration of 10 µg/mL as described in previous research [22], normalized to equivalent vesicular protein content as determined by Bradford assay. Vehicle (PBS) controls were processed in parallel under identical serum-free conditions. Following 24 h of incubation at 37 °C in 5% CO₂, cells were collected for downstream molecular analyses, including RNA extraction and western blotting.

### 2.10. RNA extraction, cDNA synthesis, and RT-qPCR analysis

Total RNA was isolated from treated MCF-7 and BNL CL.2 cells using QIAzol Lysis Reagent (Qiagen, Hilden, Germany) in combination with the GeneJET RNA Purification Kit (Thermo Fisher Scientific, USA), following the manufacturers’ instructions. RNA concentration and purity were assessed spectrophotometrically using an Infinite®200 PRO NanoQuant system (Tecan, Switzerland). For each sample, 1 μg of total RNA was reverse transcribed into cDNA using a Thermo Scientific cDNA Synthesis Kit (Cat# K1622, Thermo Scientific), according to the manufacturer’s protocol. Quantitative real-time PCR was performed using SYBR™ Green PCR Master Mix (Applied Biosystems, USA) on a StepOnePlus™ Real-Time PCR System (Applied Biosystems, CA, USA). Relative gene expression levels were calculated using the 2^ − ΔΔCt method. *GAPDH* was used as the endogenous reference gene for normalization in MCF-7 samples, whereas *Actb* was used for normalization in mouse BNL CL.2 samples. EMT-associated transcripts *CDH1* (E-cadherin), *VIM* (vimentin), and *ZEB2* were quantified in MCF-7 cells, while *Il-6, Tnf-α, Mmp-2*, and *Mmp-9* were measured in BNL CL.2 cells. Primer sequences are provided in S2 Table.

### 2.11. In-silico analysis

To investigate the protein interaction network involving FN1, VWF, SDC1, SDC2, SDC4, and Gal-3, we used the STRING database (Search Tool for the Retrieval of Interacting Genes/Proteins; version 11; http://string-db.org/; accessed 1 January 2025) [23]. For mRNA expression analysis of breast cancer tissues across various grades and stages, we compared them with normal and non-cancerous tissues using the METABRIC dataset available on the cBioPortal database (https://www.cbioportal.org/) (accessed on 1^st^ January 2025) [24]. The TNM-Plot tool (https://tnmplot.com/analysis/) (accessed on 1^st^ January 2025) [25] was employed to assess correlations between the proteome profiles of breast carcinoma and normal breast tissues. The FunRich software tool (version 3.1.3), integrated with the Vesiclepedia database (http://www.microvesicles.org/, accessed on 1^st^ January 2025) [26], was employed to perform a detailed comparative analysis of cellular components, molecular functions, biological pathways, and biological processes. Specifically, interactions were constructed among the validated proteins (FN1, SDC2, VWF, and Gal-3), as well as between these validated proteins and other proteins identified in the proteomics data. Additionally, the validated proteins and those identified in the proteomics results were analyzed for their interactions with other proteins within the proteoglycan pathway. This approach provided insights into the network relationships and functional roles of these proteins. The protein expression levels of FN1, VWF, SDC2, and Gal-3 in breast cancer tissue compared to normal breast tissue were retrieved from the Human Protein Atlas database (https://www.proteinatlas.org/) (accessed on 1^st^ January 2025) using immunohistochemistry data [27]. To assess the predictive potential of *FN1*, *VWF*, *SDC2*, and *LGALS3* in breast cancer treatment outcomes, we used the receiver operating characteristic (ROC) Plotter database (https://rocplot.com/site/treatment) (accessed on 1^st^ January 2025) [28]. ROC analysis was performed using the ROC-Plotter database to evaluate the association between gene expression and chemotherapy response. The analysis was based on the pathological complete response (pCR) dataset, representing patients who received neoadjuvant chemotherapy. Both the combined chemotherapy cohort and individual chemotherapy regimen cohorts were evaluated, including commonly used anthracycline- and taxane-based treatments (e.g., FEC, FAC, CMF, and taxane-containing regimens), as implemented within the ROC-Plotter platform. Hallmark pathway enrichment analysis was conducted using the https://cancerhallmarks.com/ platform [29]. Drug–Gene Interaction Database (DGIdb) (https://dgidb.org; accessed 12 April 2025) was queried to identify drugs interacting with FN1, VWF, SDC2, and LGALS3. The interaction score for each drug–gene pair was obtained directly from DGIdb (database-calculated metric based on supporting evidence and specificity components) and reported without modification. In DGIdb, the interaction score is calculated as the product of an evidence score (publication count + source count) and two specificity terms (relative drug specificity and relative gene specificity), where each specificity term reflects the ratio of the average number of known partners across all drugs/genes to the number of known partners for the specific drug or gene in the interaction [30].

### 2.12. Statistical analysis

Data analysis was conducted using SPSS software (version 27). The normality of the data was assessed through skewness and kurtosis, followed by the application of parametric tests for normally distributed data. Student’s t-test was used to evaluate differences between two groups. For non-parametric analysis, a Chi-square test was used to examine clinicopathological data. A one-way analysis of variance (ANOVA) was applied to assess differences among more than two groups and post hoc Tukey’s test was used to identify specific group differences. Pearson and Spearman correlation tests were used to evaluate relationships between variables. Protein identifications were filtered using a false discovery rate (FDR) threshold of 1% at the protein level based on a decoy-database approach to ensure high-confidence protein identification. Results are presented as mean ± SEM, and *P <* 0.05 was considered statistically significant. Graphs were generated using GraphPad Prism 8 (version 8). FunRich software tool (version 3.1.3) was used to make Venn diagrams, functional annotations, and protein-protein interaction networks.

## 3. Results

### 3.1. Clinicopathologic features for breast cancer patients

Breast cancer patients enrolled in the study were obese (with BMI > 30) and had not received neoadjuvant chemotherapy prior to sample collection. Age-matched obese individuals were recruited as control subjects. All relevant clinicopathologic features of breast cancer patients are summarized in Table 1.

**Table 1:** Clinicopathologic characteristics of normal controls and breast cancer patients.

Characteristic	Breast cancer patients (n = 76)	Normal controls (n = 36)	*P* value
**Age (years)**			^*a*^ *P > 0.05*
Range	34-79	36-79	
Mean ± SEM	58.43 ± 1.32	54.31 ± 1.88	
<50	20 (26.4%)	14 (38.78%)	^*b*^*P* > 0.05
≥50	55 (72.6%)	22 (61.22%)	
NA	1 (1.32%)	–	
**Body Mass Index (BMI)** Range Mean ± SEM			^*a*^ *P > 0.05*
	30.18–48.83	30.66–48	
	36.52 ± 0.598	37.01 ± 0.818	
**Family history, n (%)**		–	
Yes	11 (14.47%)	–	
No	64 (84.21%)	–	
NA	1 (1.32%)	–	
**Laterality, n (%)**		–	
Bilateral	3 (3.95%)	–	
Right	37 (48.68%)	–	
Left	36 (47.37%)	–	
**NA**		–	
**Tumor size (cm), n (%)**		–	
< 4	57 (75%)	–	
≥ 4	19 (25%)	–	
**NA**	–	–	
**Tumor grade, n (%)**		–	
Grade I	4 (5.26%)	–	
Grade II	62 (81.58%)	–	
Grade III	9 (11.84%)	–	
NA	1 (1.32%)	–	
**ER, n (%)**		–	
Negative	4 (5.26%)	–	
Positive	72 (94.74%)	–	
NA	–	–	
**PR, n (%)**		–	
Negative	4 (5.26%)	–	
Low positive	7 (9.21%)	–	
Positive	65 (85.53%)	–	
NA	–	–	
**HER2, n (%)**		–	
Negative	64 (84.21%)	–	
Equivocal (non-amplified)	12 (15.79%)	–	
Positive	–	–	
**NA**	–	–	
**Stages, n (%)**		–	
I A	23 (30.26%)	–	
I B	1 (1.32%)		
II A	17 (22.37%)	–	
II B	15 (19.74%)	–	
III A	11 (14.47%)	–	
III B	2 (2.63%)	–	
III C	7 (9.21%)	–	
NA	–	–	
**Lymph node status, n**		–	
N0	40 (52.63%)	–	
N1	18 (23.68%)	–	
N2	11 (14.47%)	–	
N3	7 (9.21%)	–	
NA		–	
**Tumor size, n (%)**		–	
T1	31 (40.79%)	–	
T2	39 (51.32%)	–	
T3	4 (5.26%)	–	
T4	2 (2.63%)	–	
NA	–	–	
**Molecular subtype n (%)**			
LumA	31 (40.79%)	–	
LumB	31 (40.79%)	–	
TNBC	4 (5.26%)	–	
NA	10 (13.16%)	–	
**Ki-67% n (%)**			
Low Ki-67 (<20%)	32 (42.11%)	–	
High Ki-67 (≥ 20%)	25 (32.89%)	–	
NA	19 (25.00%)	–	

^a^: Student’s t-test P value, ^b^: Chi-square test P value, BMI: Body mass index, ER: estrogen-receptor, PR: progesterone-receptor, HER2: Human epidermal growth factor-2, and SEM: standard error of mean.

### 3.2. Confirmation of successful enrichment of plasma-derived small-EVs

**Fig 1:**
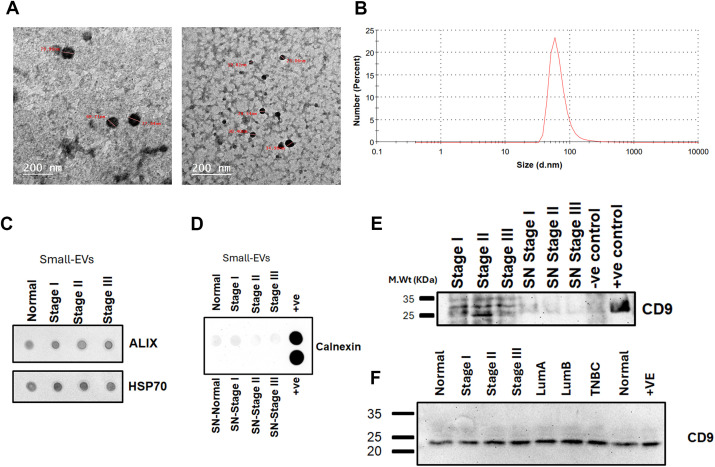
Characterization of plasma-derived small extracellular vesicles (small-EVs). **(A)** High-resolution transmission electron microscopy (HR-TEM) images (scale bars: 200 nm). **(B)** Dynamic light scattering (DLS) size distribution. **(C)** Dot blot of ALIX, heat shock protein 70 (HSP70). **(D)** Dot blot of calnexin (negative extracellular vesicle marker) in normal controls and breast cancer stage I–III small-EVs and matched supernatants (SN). **(E)** Western blot of CD9 in stage I–III small-EVs and matched SN. **(F)** CD9 western blot across normal controls and breast cancer groups (stages I–III; Luminal A (LumA), Luminal B (LumB), triple-negative breast cancer (TNBC)). Positive control: pooled breast cancer cell-line lysate; negative control: bovine serum albumin (BSA) (Wu et al., 2007). The uncropped full-length blot is provided in Supplementary S1_raw_images file.

The successful enrichment of plasma-derived small-EVs was validated using multiple complementary approaches (Fig 1A–1E). HR-TEM confirmed the presence of rounded membrane-bound vesicles with morphology and size consistent with small-EVs (<200 nm) (Fig 1A and S1 Fig). In parallel, DLS analysis demonstrated a predominant vesicle population within the expected small-EVs range, with a major peak at 58.77 nm. The measured polydispersity index (PDI = 0.390) indicated moderate size dispersion, supporting the enrichment of a small-sized vesicular population (Fig 1B).

To further confirm EV enrichment, dot blot and western blot analyses were performed using established small EV markers CD9, ALIX, and HSP70. Dot blot analysis showed that ALIX and HSP70 were consistently detected in small-EVs enriched fractions across normal control and breast cancer stages (I–III) (Fig 1C), whereas the endoplasmic reticulum marker Calnexin (negative EV marker) was not detected, indicating limited detectable contamination with cellular debris and non-vesicular components (Fig 1D). Western blot analysis further validated CD9 enrichment in the isolated small-EVs fractions from breast cancer stages I–III, with minimal to absent detection in the corresponding post-precipitation supernatants (SN) and negative controls, supporting enrichment of small-EVs (Fig 1E). In addition, CD9 expression showed consistent detection across controls, breast cancer stages, and molecular subtypes (LumA, LumB, and TNBC) (Fig 1F).

### 3.3. Identification of proteomic profiling of plasma-derived small-EVs isolated from breast cancer patients

**Fig 2:**
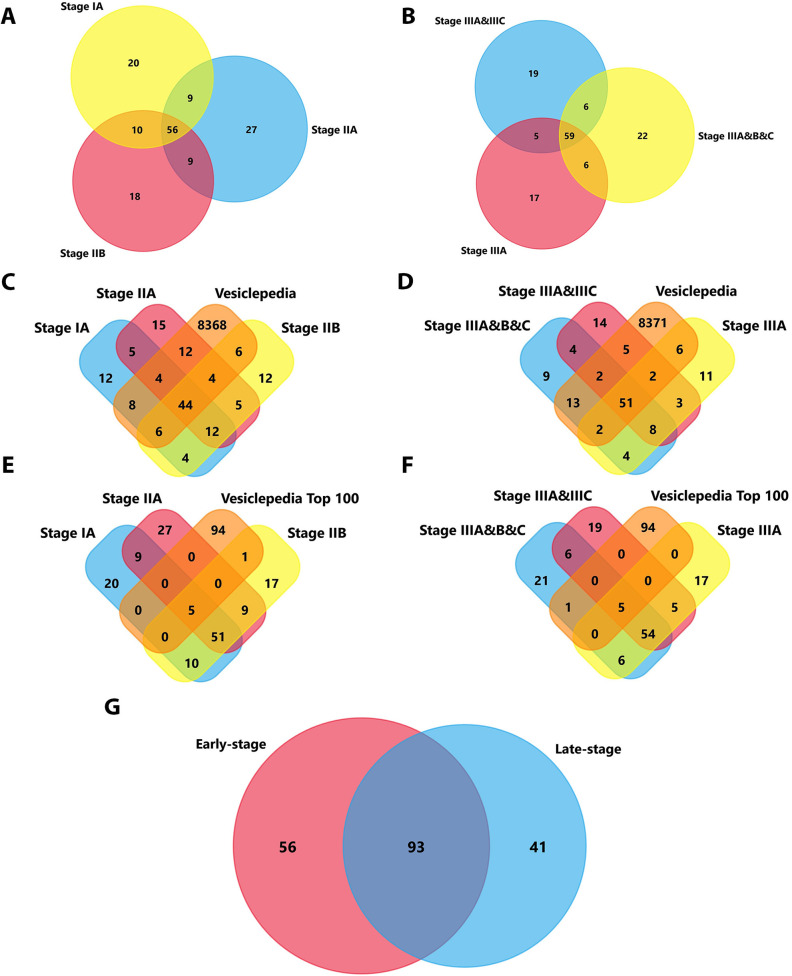
Proteomic profiling of plasma-derived small extracellular vesicles (small-EVs) from breast cancer patients. **(A–B)** Venn diagrams showing overlap of identified small-EVs proteins across early-stage subgroups (stages IA, IIA, IIB) (A) and late-stage subgroups (stages IIIA, IIIB, IIIC; and combined subsets as indicated) **(B)**. **(C–D)** Overlap of stage-stratified small-EVs protein lists with the Vesiclepedia extracellular vesicle protein database for early-stage (C) and late-stage (D) groups. **(E–F)** Overlap of the top 100 extracellular vesicle proteins (“Vesiclepedia Top 100”) with small-EVs protein lists (E) early-stage and (F) late-stage. **(G)** Summary Venn diagram comparing early-stage vs late-stage small-EVs proteomes. Venn diagrams were generated using FunRich (version 3.1.3) integrated with the Vesiclepedia database (accessed 1 January 2025) [26].

Discovery proteomic profiling was performed on a screening breast cancer cohort, including stage I (n = 5), stage II (n = 10), and stage III (n = 10) patients. Comparative Venn diagram analysis revealed distinct and overlapping proteomic signatures between early-stage breast cancer (IA, IIA, IIB) and late-stage breast cancer (IIIA, IIIB, IIIC), as illustrated in Fig 2A, 2B. The identified small-EVs proteins were cross-referenced with Vesiclepedia, a curated database of reported EVs proteins. Small-EVs from early-stage BC samples demonstrated a substantial overlap, with 84 proteins matching entries in Vesiclepedia (Fig 2C). Similarly, late-stage samples showed enrichment with 81 overlapping proteins (Fig 2D), further supporting the EV-enriched nature of the samples.

A focused analysis of the Vesiclepedia Top 100 EV proteins revealed substantial overlap between early- and late-stage samples. Specifically, five proteins were shared between early- and late-stage samples within the Vesiclepedia Top 100 overlap, namely A2M, ALB, FN1, GSN, and LGALS3 BP. In addition, CLTC was uniquely represented in early-stage samples, whereas ANXA1 was uniquely represented in late-stage samples (Fig 2E, 2F). Further stratification showed that 56 proteins (29.3%) were uniquely associated with early-stage breast cancer, 41 proteins (22%) were exclusive to late-stage disease, and 93 proteins (48.7%) were common across both groups (Fig 2G). The common and stage-specific protein sets are provided in S3 Table. All raw proteomics files are provided in the S1-S6 Files.

### 3.4. Functional annotation and PPI network of identified small-EVs proteins

**Fig 3:**
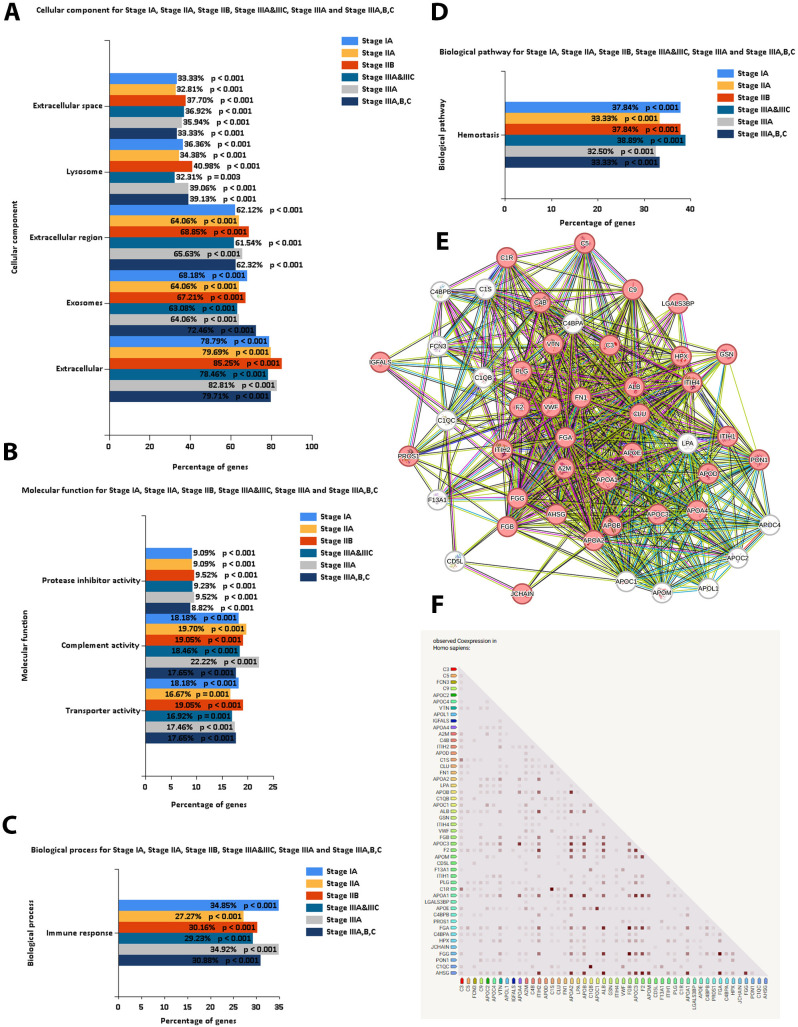
Functional annotation and interaction analyses of plasma-derived small extracellular vesicle (small-EVs) proteins. Identified small-EVs proteins were mapped to gene symbols and analyzed at the gene level (x-axis: percentage of mapped genes). **(A)** Cellular component enrichment. **(B)** Molecular function enrichment. **(C)** Biological process enrichment. **(D)** Biological pathway enrichment. **(E)** Protein–protein interaction (PPI) network generated using Search Tool for the Retrieval of Interacting Genes/Proteins (STRING; version 11; http://string-db.org/; accessed 1 January 2025); red nodes indicate proteins annotated as “extracellular exosome” in STRING. **(F)** STRING co-expression matrix for the indicated proteins.

The identified small EV proteins were mapped to their corresponding gene symbols, and enrichment analysis conducted at the gene level (Fig 3A–3F) revealed significant enrichment across several biological categories. Cellular component analysis highlighted a predominant localization of proteins within the extracellular space, lysosomes, extracellular region, and exosomes (Fig 3A), confirming the small-EVs nature of the isolated vesicles. Molecular function analysis demonstrated enrichment in protease inhibitor activity, complement binding, and transporter activity (Fig 3B). In the biological process category, a strong association was observed with immune response modulation (Fig 3C). Additionally, pathway enrichment analysis revealed significant involvement in hemostasis-related pathways, emphasizing cancer-associated coagulation processes (Fig 3D). Among the proteomic profiles, a set of 48 common proteins were identified across all stages of breast cancer (S3 Table). Within this subset, FN1, VWF, and PROS1 emerged as prominent candidates based on their consistent expression across disease stages and established functional relevance in cancer biology [31–33]. Further PPI network and co-expression analysis demonstrated that FN1 and VWF function as hub proteins, suggesting their central roles in key signaling pathways related to breast cancer progression (Fig 3E, 3F). In summary, the consistent detection of FN1, VWF, and PROS1 in plasma-derived small-EVs proteomes suggests that these proteins may represent candidate biomarkers associated with disease progression.

### 3.5. Validation of FN1, VWF, and PROS1 across breast cancer stages and molecular subtypes

**Fig 4:**
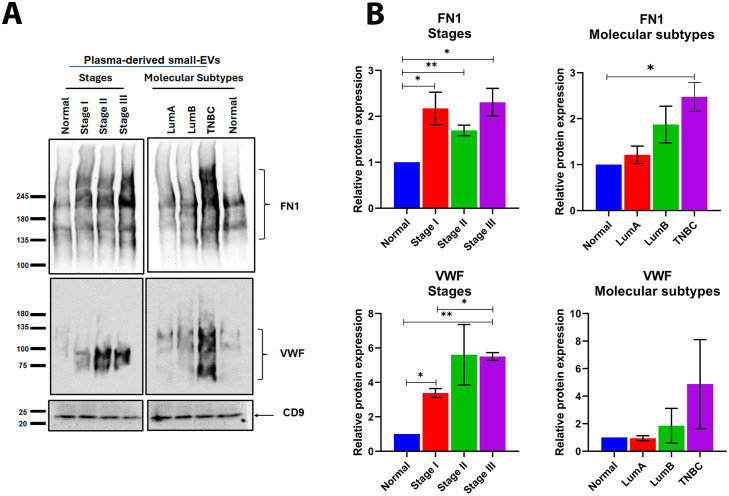
Validation of Fibronectin 1 (FN1) and von Willebrand factor (VWF) in plasma-derived small extracellular vesicles (small-EVs). **(A)** Representative western blots of small-EVs from normal controls and breast cancer patients stratified by clinical stages: stage I (n = 19), stage II (n = 22), and stage III (n = 10) and molecular subtype (Luminal A (LumA) (n = 31), Luminal B (LumB) (n = 31), triple-negative breast cancer (TNBC) (n = 4)) probed for FN1, VWF, and CD9. **(B)** Protein level quantification of FN1 and VWF across stages (left) and molecular subtypes (right), shown as relative protein expression. Data are presented as mean ± SEM (n ≥ 2). Statistical significance was assessed using Student’s t-test (*P < 0.05 and *P < 0.001). The uncropped full-length blot is provided in Supplementary S1_raw_images file.

To validate the proteomic findings, plasma-derived small-EVs were analyzed by western blotting in independent validation cohort across stage I (n = 19), stage II (n = 22), and stage III (n = 10) and molecular subtypes, including LumA, LumB, and TNBC. Western blot analysis of plasma-derived small-EVs proteins revealed a consistent presence of FN1 across all stages and molecular subtypes. Notably, FN1 expression was significantly elevated in patients with advanced-stage disease and in the TNBC subtype compared to normal controls (*P <* 0.05) (Fig 4A–4C).

Similarly, VWF was highly expressed in small-EVs from breast cancer patients, while it was undetectable or minimally expressed in control samples. The highest levels of VWF were observed in patients with advanced-stage disease (stage III), with statistically significant differences compared to controls (*P <* 0.05). In contrast, PROS1 did not exhibit a distinct or stage-dependent expression pattern. It was detected in small-EVs from both normal control individuals and breast cancer patients without variations (S2 Fig).

### 3.6. Interaction-guided validation of SDC2 and Gal-3 in plasma-derived small-EVs and MV-enriched EVs

**Fig 5:**
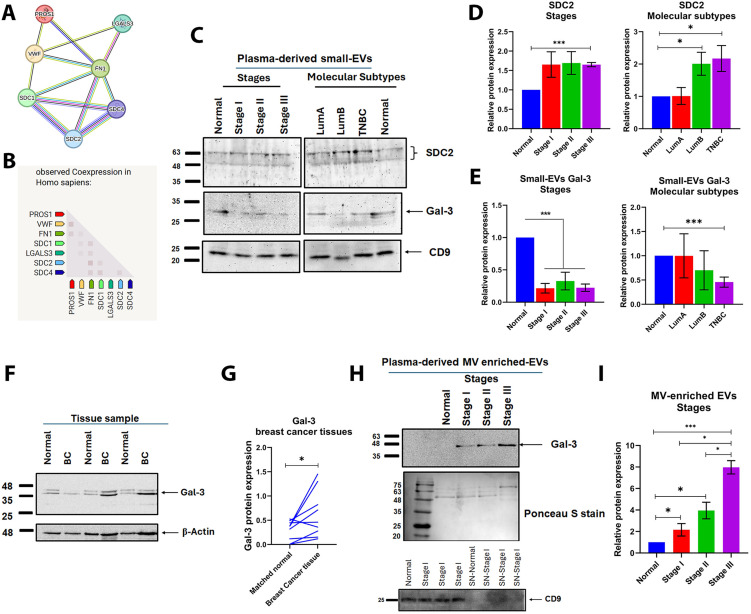
Validation of Syndecan-2 (SDC2) and Galectin-3 (Gal-3) in plasma-derived small extracellular vesicles (small-EVs), normal and breast carcinoma tissues, and/or microvesicle-enriched extracellular vesicles (MV-enriched EVs). **(A)** Search Tool for the Retrieval of Interacting Genes/Proteins (STRING; version 11; http://string-db.org/; accessed 1 January 2025) protein–protein interaction network for Fibronectin 1 (FN1), von Willebrand factor (VWF), Protein S (PROS1), Syndecan-1 (SDC1), SDC2, Syndecan-4 (SDC4), and LGALS3 (Gal-3). **(B)** STRING co-expression matrix for the same proteins in Homo Sapiens. **(C)** Representative western blots of plasma-derived small-EVs from normal controls and breast cancer patients stratified by clinical stages: stage I (n = 19), stage II (n = 22), and stage III (n = 10) and molecular subtype (Luminal A (LumA) (n = 31), Luminal B (LumB) (n = 31), triple-negative breast cancer (TNBC) (n = 4)) probed for SDC2, Gal-3, and CD9. **(D–E)** Protein level quantification of small-EVs SDC2 (D) and small-EVs Gal-3 (E) across stages (left) and molecular subtypes (right), shown as relative protein expression **(F)** Representative western blot of Gal-3 in breast carcinoma (BC) tissues and matched adjacent normal tissues; beta-actin (β-actin) is used as a loading control. **(G)** Paired protein level quantification of tissue Gal-3 in matched samples. **(H)** Representative western blot of Gal-3 in plasma-derived MV-enriched EVs across normal controls and stages I–III; Ponceau S staining is shown as a total-protein loading control; CD9 is shown as an EV marker in MV-enriched EVs and corresponding post-pelleting supernatants (SN). **(I)** Protein level quantification of MV-enriched EVs Gal-3 across stages. Data are presented as mean ± SEM (n ≥ 2). Statistical significance was assessed using Student’s t-test (*P < 0.05 and *P < 0.001). The uncropped full-length blot is provided in Supplementary S1_raw_images file.

FN1, the SDC family, VWF, and Gal-3 are known to play pivotal roles in breast cancer progression through their interactions with the tumor microenvironment (TME) [34,35]. For example, FN1 interacts with SDC2, initiating downstream signaling cascades involved in focal adhesion, which regulate cytoskeletal remodeling and promote tumor cell migration and invasion [36]. VWF has also been implicated in tumor angiogenesis and metastasis [32], while Gal-3 contributes to tumor cell adhesion, immune evasion, and chemoresistance [37]. These findings suggest that these proteins may act as interconnected mediators in breast cancer progression. To explore their functional relationships, a STRING-based PPI network analysis was performed (Fig 5A, 5B). The analysis revealed that FN1, VWF, and PROS1 interact with SDCs (SDC1, SDC2, SDC4) and Gal-3, supporting a potential co-regulatory role in tumor-associated signaling.

To validate these interactions, we examined the expression of SDC1, SDC2, SDC4, and Gal-3 in plasma-derived small-EVs from breast cancer patients. Western blot analysis confirmed significantly higher SDC2 expression in small-EVs from stage III (P < 0.001), LumB (*P <* 0.05), and TNBC patients *(P <* 0.05) compared to normal controls (Fig 5C, 5D). In contrast, SDC1 was not detected in small-EVs from either normal controls or breast cancer patients, while SDC4 was present in both groups without differences (S2 Fig). Interestingly, Gal-3 was detected in small-EVs from normal controls but was significantly reduced in those derived from breast cancer patients (*P <* 0.001, Fig 5C–5E). However, analysis of matched samples, consisting of paired primary tumor tissues and their corresponding adjacent normal breast tissues collected from the same patients, revealed a contrasting expression pattern. Western blot analysis showed significantly elevated Gal-3 levels in tumor tissues compared to their paired adjacent normal tissues (*P <* 0.05, Fig 5F, 5G).

Gal-3 associates with the plasma membrane via extracellular binding to surface glycoconjugates or intracellular accumulation at the cytoplasmic face during unconventional secretion [37] and is linked to plasma-membrane blebbing/vesicular budding and low-density vesicle fractions, consistent with a bias toward plasma membrane–derived EVs (microvesicles/ectosomes) in some contexts [38]. Because Gal-3 lacks a classical signal sequence, it is secreted through nonclassical pathways [39], including direct plasma-membrane transfer and release within plasma membrane–derived microvesicles [40]. Accordingly, we assessed Gal-3 in plasma-derived MV-enriched EVs across breast cancer stages. The clinicopathologic features of the patients included in the MV-enriched EV analysis are summarized in S4 Table. Gal-3 was significantly increased in MV-enriched EVs of plasma of breast cancer in staging-dependent manner (stage I and II (P < 0.05), stage III (*P* < 0.001) with no detectable levels in their counterpart controls. Additional validation of CD9 as an EV marker showed enrichment in MV-enriched EVs and absence in their corresponding post-pelleting supernatants (Fig 5H, 5I). Collectively, Gal-3 showed compartment-specific EV distribution, with reduced levels in small-EVs but enrichment in tumor tissues and MV-enriched EVs during breast cancer progression.

### 3.7. Plasma TNBC-derived small-EVs affects expression of EMT-related factors in MCF7 cells and of inflammatory- and matrix-remodeling-related genes in BNL CL.2 cells

**Fig 6:**
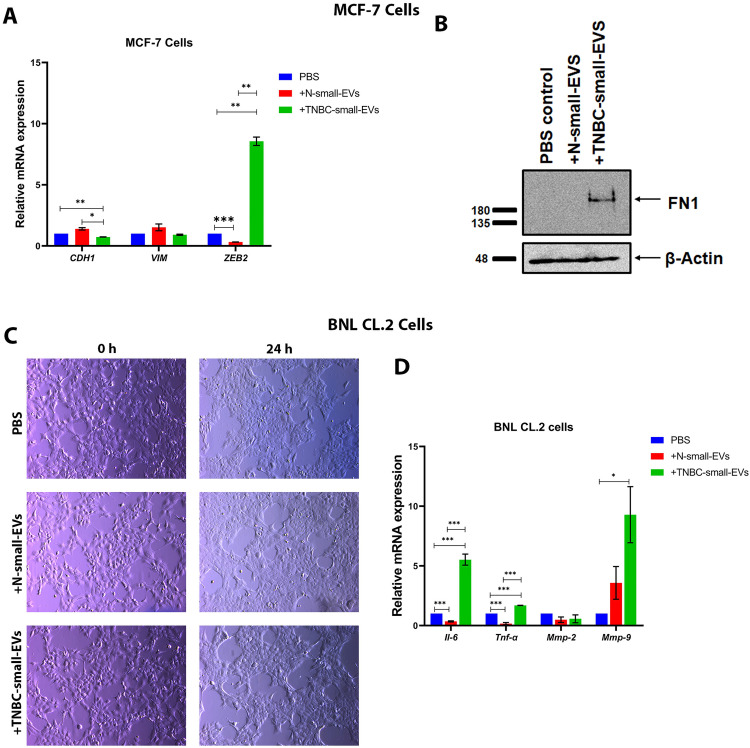
Effects of plasma-derived small extracellular vesicles (small-EVs) on MCF-7 and BNL CL.2 cells. MCF-7 human breast cancer cells and BNL CL.2 mouse liver cells were treated with phosphate-buffered saline (PBS), normal control–derived small-EVs (N-small-EVs), or triple-negative breast cancer–derived small-EVs (TNBC-small-EVs) (10 µg/mL, 24 h). (A) Quantitative polymerase chain reaction (qPCR) analysis of epithelial–mesenchymal transition (EMT)-related transcripts (CDH1, VIM, and ZEB2) in MCF-7 cells. (B) Western blot of Fibronectin 1 (FN1) in MCF-7 cells; beta-actin (β-actin) as a loading control. (C) Phase-contrast micrographs of BNL CL.2 cells at 0 h and 24 h under the indicated treatments (200×). (D) qPCR analysis of interleukin-6 (Il-6), tumor necrosis factor-alpha (Tnf-α), matrix metalloproteinase-2 (Mmp-2), and matrix metalloproteinase-9 (Mmp-9) in BNL CL.2 cells. Data are presented as mean ± SEM (n ≥ 2). Statistical significance was assessed using Student’s t-test (*P < 0.05, **P < 0.01, and ***P < 0.001). The uncropped full-length blot is provided in Supplementary S1_raw_images file.

To investigate impact of circulating plasma-derived small-EVs on expression levels of EMT-related markers in epithelial MCF7 cells, the cells were treated with either an equivalent dose of plasma-derived small-EVs (10 μg/mL) isolated from normal controls (N-small-EVs), TNBC patients (TNBC-small-EVs), or equal volume of PBS as a vehicle control. Exposure of MCF-7 cells to TNBC-small-EVs resulted in a significant reduction of *CDH1* expression (encoding for E-cadherin, *P <* 0.01) expression, accompanied by a significant induction (*P <* 0.01), of its transcriptional suppressor *ZEB2* expression [41] consistent with a shift toward an EMT-like transcriptional state (Fig 6A). Intriguingly, immunoblotting demonstrated increased FN1 protein expression in MCF-7 cells subjected to TNBC-small-EVs (Fig 6B). *VIM* mRNA expression has not been altered upon treatment with TNBC-small-EVs in MCF7 cells.

Since liver is a known metastatic organ for breast cancer cells [42] and EVs are implicated in preparation of the premetastatic niche via intercellular communication [43], we next assessed influence of plasma TNBC-derived small-EVs on BNL CL.2 normal liver cells. The cells were treated with 10 μg/mL N-small-EVs or TNBC-small-EVs, beside PBS as vehicle controls (Fig 6C) and expression levels of inflammatory- and ECM remodeling-related genes *Il-6*, *Tnf-α*, *Mmp-2* and *Mmp-9* were quantified. qPCR results demonstrated a significant increased *Il-6* (*P* < 0.001), *Tnf-α (P <* 0.001) and *Mmp-9* (*P <* 0.05) transcript levels following TNBC-small-EVs treatment compared to vehicle controls (Fig 6D), whereas *Mmp-2* mRNA expression did not alter. Collectively, these findings suggest that plasma-derived small-EVs, particularly those derived from TNBC patients, can promote EMT-associated transcriptional changes in low aggressive luminal-like MCF7 breast cancer cells and trigger inflammatory and matrix remodeling programs in BNL CL.2 liver cells, consistent with their mechanistic role in microenvironmental conditioning and metastatic niche priming.

### 3.8. Gene and protein expression analysis of FN1, VWF, SDC2, and Gal-3 across breast cancer molecular subtypes using online databases

To provide differential expression patterns of FN1, VWF, SDC2, and Gal-3 in breast cancer, we integrated METABRIC mRNA data with protein evidence from HPA immunohistochemistry and UALCAN/CPTAC proteomics and used HPA single-cell RNA profiles to define the major cellular sources of each marker.

METABRIC mRNA analysis demonstrated subtype- and clinicopathologic feature-specific expression of *FN1*, *VWF*, *SDC2*, and *LGALS3* (Gal-3) across breast cancer clinicopathologic variables. *FN1* was significantly upregulated in TNBC and HER2-enriched tumors compared with LumA/LumB (*P <* 0.01) and correlated with higher histological grade (*P <* 0.001). In contrast, *VWF* was significantly reduced in tumors versus normal tissue (*P <* 0.001) and showed higher expression in LumA/LumB compared with TNBC/HER2-enriched (*P <* 0.001). *SDC2* expression was highest in HER2-enriched tumors compared with LumA/LumB/TNBC (*P <* 0.001) and increased in Stage II versus Stage I (*P <* 0.05). *LGALS3* was elevated in HER2-enriched and TNBC relative to LumA/LumB (*P <* 0.05) and was higher in lymph node-positive patients (*P <* 0.05) and in those receiving chemotherapy (*P <* 0.001) (S3 Fig).

**Fig 7:**
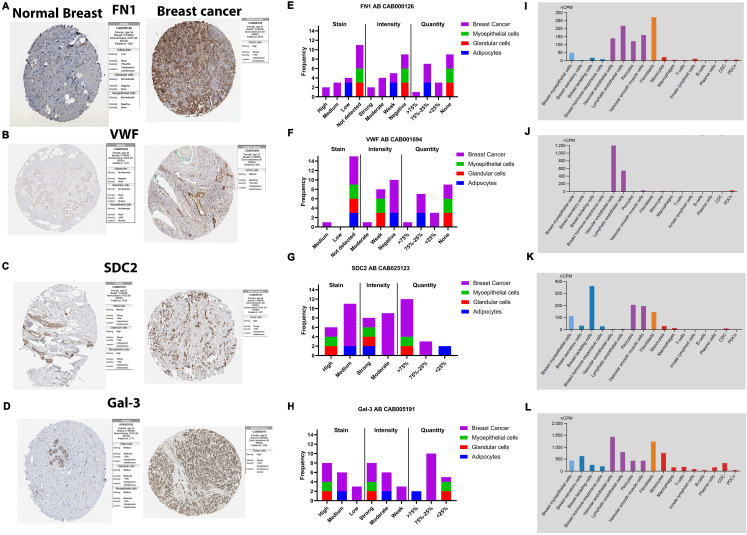
Human Protein Atlas validation of Fibronectin 1 (FN1), von Willebrand factor (VWF), Syndecan-2 (SDC2), and Galectin-3 (Gal-3) in breast tissues and cell types. **(A–D)** Representative immunohistochemistry (IHC) images of normal breast versus breast cancer tissues for FN1 **(A)**, VWF **(B)**, SDC2 **(C)**, and Gal-3 **(D)**. **(E–H)** Human Protein Atlas (HPA) IHC summary plots showing staining, intensity, and quantity across annotated cell types (antibody IDs as indicated) for FN1 **(E)**, VWF **(F)**, SDC2 **(G)**, and Gal-3 **(H)**. **(I–L)** HPA single-cell RNA expression profiles shown as normalized counts per million (nCPM) across breast cell populations for FN1 **(I)**, VWF **(J)**, SDC2 **(K)**, and LGALS3 **(L)**. Data were retrieved from the Human Protein Atlas database (https://www.proteinatlas.org/; accessed 1 January 2025).

Protein-level evaluation supported these transcriptomic trends and highlighted compartment-specific expression. HPA IHC showed stronger tumor-associated staining for FN1 (predominantly extracellular/cytoplasmic), while VWF staining was largely confined to vascular/endothelial structures, consistent with its vascular localization. SDC2 displayed increased staining in breast carcinoma compared with normal tissue, and Gal-3 showed stronger tumor-associated staining with cytoplasmic and nuclear distribution. HPA single-cell RNA further clarified the dominant cellular sources: FN1 was highest in stromal/fibroblast-like populations (supporting ECM remodeling), VWF was enriched in endothelial cells, SDC2 showed enrichment in stromal/structural compartments (supporting cell–matrix interactions), and LGALS3 (Gal-3) was prominent in immune (myeloid/macrophage-lineage) compartments, indicating that Gal-3 signals in bulk tissue may reflect both tumor-associated expression and immune infiltration (Fig 7).

Finally, UALCAN/CPTAC proteomics confirmed differential protein abundance between normal breast tissues and primary tumors, with FN1 (*P <* 0.001) and SDC2 (*P <* 0.05) being significantly elevated in tumors and across stage/subtype stratifications. In contrast, VWF protein was significantly lower in tumors versus normal tissues (*P <* 0.001), consistent with its vascular-restricted distribution, and Gal-3 protein was also reduced in tumors (*P <* 0.001), suggesting that its biological relevance may be driven by compartment-specific tumor/immune niches rather than uniform bulk-tumor protein elevation (S4 Fig). Notably, the lower bulk-tumor Gal-3 signal observed in CPTAC contrasts with the higher Gal-3 levels detected in our paired tissue immunoblots, potentially reflecting differences in cohort composition, tissue compartmentalization, and limited sample size of our tissue samples.

### 3.9. Interaction networks and hallmark pathway enrichment analysis of FN1, VWF, SDC2, and Gal-3

**Fig 8:**
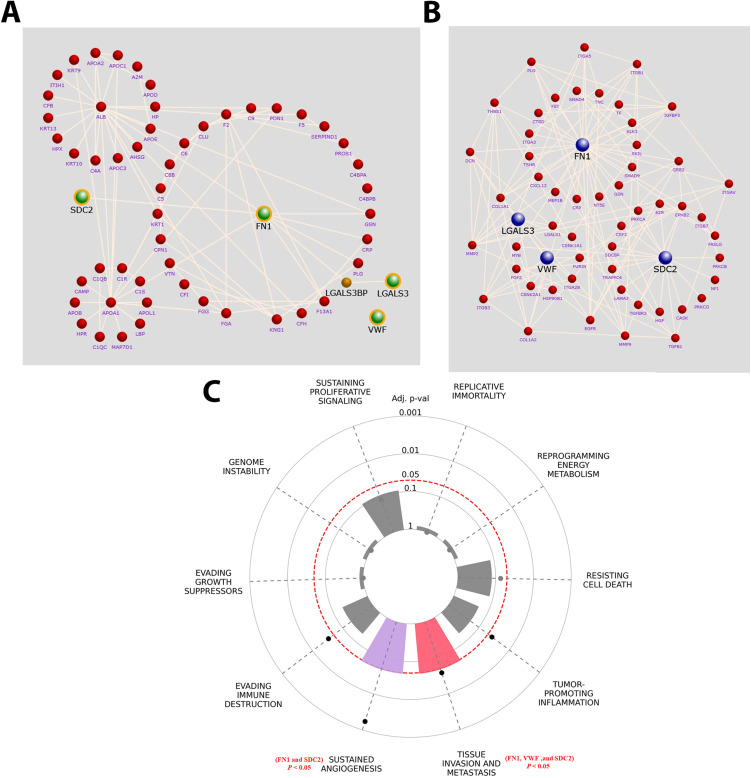
Interaction networks and hallmark pathway enrichment of Fibronectin 1 (FN1), von Willebrand factor (VWF), Syndecan-2 (SDC2), and Galectin-3 (Gal-3) in breast cancer. **(A)** FunRich interaction network showing FN1, VWF, SDC2, and Gal-3 with proteomics-identified interacting proteins. **(B)** FunRich proteoglycan pathway interaction network for FN1, VWF, SDC2, and Gal-3. **(C)** Cancer hallmarks enrichment plot showing associations with tissue invasion and metastasis (adjusted P < 0.05; FN1, VWF, SDC2) and sustained angiogenesis (adjusted P < 0.05; FN1, SDC2).

An interaction network was generated to define relationships among FN1, VWF, SDC2, Gal-3, and other shared proteins identified by small-EVs proteomic profiling. The network showed extensive interconnectivity and highlighted FN1 as a central hub coordinating multiple molecular interactions (Fig 8A). Pathway-focused mapping further identified the proteoglycan signaling pathway as highly enriched, with FN1, VWF, SDC2, and Gal-3 displaying strong connectivity to several pathway components, supporting their involvement in tumor-associated ECM remodeling and signaling (Fig 8B). Finally, CancerHallmarks.com analysis linked these proteins to the ten canonical cancer hallmarks and showed significant enrichment in pathways related to sustained angiogenesis and tissue invasion and metastasis (adjusted *P <* 0.05; Fig 8C), reinforcing their potential roles in breast cancer progression.

### 3.10. Predictive value of *FN1*, *VWF*, *SDC2*, and *LGALS3* in response to chemotherapy and anti-HER2 therapies

**Fig 9:**
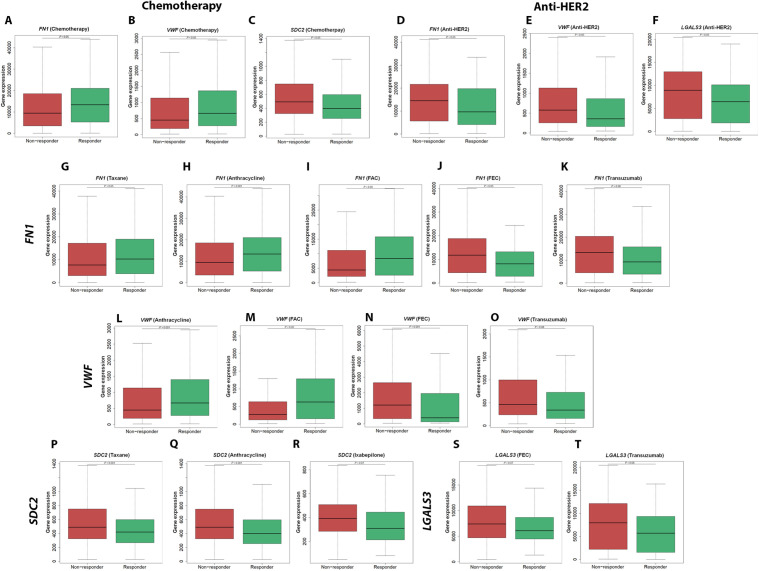
Therapy-response–associated gene expression of Fibronectin 1 (*FN1*), von Willebrand factor (*VWF*), Syndecan-2 (*SDC2*), and *LGALS3* (Galectin-3; Gal-3) using ROC Plotter. Boxplots show gene expression in non-responders versus responders for (A–C) chemotherapy (non-responders, n = 1100; responders, n = 532): *FN1* **(A)**, *VWF* **(B)**, *SDC2* **(C)**; and (D–F) anti–human epidermal growth factor receptor 2 (anti-HER2) therapy (non-responders, n = 122; responders, n = 95): *FN1* **(D)**, *VWF* **(E)**, *LGALS3* **(F)**. Regimen-specific analyses are shown for *FN1*: taxane (G; non-responders, n = 842; responders, n = 371), anthracycline (H; non-responders, n = 1098; responders, n = 528), FAC (I; non-responders, n = 186; responders, n = 62), FEC (J; non-responders, n = 219; responders, n = 84), and trastuzumab (K; non-responders, n = 99; responders, n = 87); for *VWF*: anthracycline (L; non-responders, n = 1098; responders, n = 528), FAC (M; non-responders, n = 186; responders, n = 62), FEC (N; non-responders, n = 219; responders, n = 84), and trastuzumab (O; non-responders, n = 99; responders, n = 87); for *SDC2*: taxane (P; non-responders, n = 842; responders, n = 371), anthracycline (Q; non-responders, n = 1098; responders, n = 528), and ixabepilone (R; non-responders, n = 31; responders, n = 105); and for *LGALS3*: FEC (S; non-responders, n = 219; responders, n = 84) and trastuzumab (T; non-responders, n = 99; responders, n = 87). Data were retrieved from ROC Plotter (https://rocplot.com/site/treatment; accessed 1 January 2025); probe sets: 212464_s_at (*FN1*), 202112_at (*VWF*), 212157_at (*SDC2*), and 208949_s_at (*LGALS3*).

**Fig 10:**
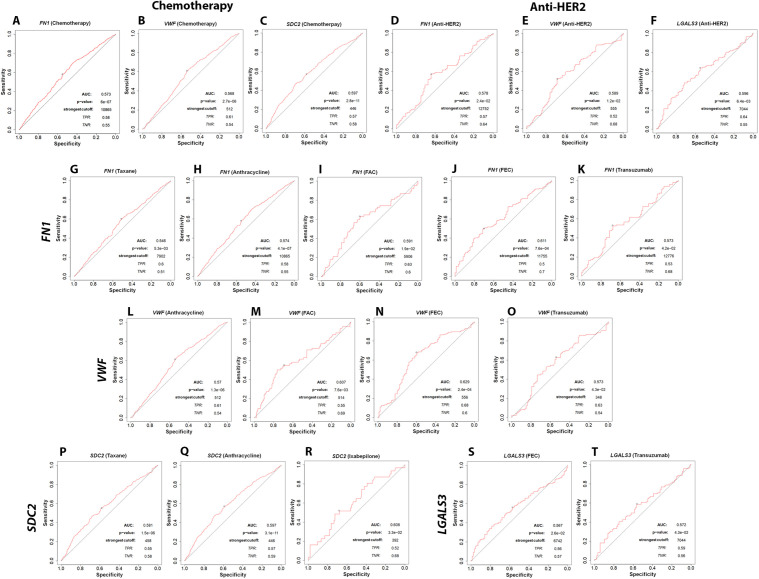
Receiver operating characteristic (ROC) analysis of therapy-response prediction for Fibronectin 1 (*FN1*), von Willebrand factor (*VWF*), Syndecan-2 (*SDC2*), and *LGALS3* (Galectin-3; Gal-3) using ROC Plotter. ROC curves compare responders versus non-responders for (A–C) chemotherapy (non-responders, n = 1100; responders, n = 532): *FN1* **(A)**, *VWF* **(B)**, *SDC2* **(C)**; and (D–F) anti–human epidermal growth factor receptor 2 (anti-HER2) therapy (non-responders, n = 122; responders, n = 95): *FN1* **(D)**, *VWF* **(E)**, *LGALS3* **(F)**. Regimen-specific ROC analyses are shown for *FN1*: taxane (G; non-responders, n = 842; responders, n = 371), anthracycline (H; non-responders, n = 1098; responders, n = 528), FAC (I; non-responders, n = 186; responders, n = 62), FEC (J; non-responders, n = 219; responders, n = 84), and trastuzumab (K; non-responders, n = 99; responders, n = 87); for *VWF*: anthracycline (L; non-responders, n = 1098; responders, n = 528), FAC (M; non-responders, n = 186; responders, n = 62), FEC (N; non-responders, n = 219; responders, n = 84), and trastuzumab (O; non-responders, n = 99; responders, n = 87); for *SDC2*: taxane (P; non-responders, n = 842; responders, n = 371), anthracycline (Q; non-responders, n = 1098; responders, n = 528), and ixabepilone (R; non-responders, n = 31; responders, n = 105); and for *LGALS3*: FEC (S; non-responders, n = 219; responders, n = 84) and trastuzumab (T; non-responders, n = 99; responders, n = 87). Data were retrieved from ROC Plotter (https://rocplot.com/site/treatment; accessed 1 January 2025); probe sets: 212464_s_at (*FN1*), 202112_at (*VWF*), 212157_at (*SDC2*), and 208949_s_at (*LGALS3*).

To explore whether *FN1*, *VWF*, *SDC2*, and *LGALS3* expression is associated with treatment response in breast cancer, we analyzed responder vs non-responder expression patterns and ROC curves using the ROC Plotter database (https://rocplot.com/site/treatment) (identifiers: 212464_s_at/FN1, 202112_at/VWF, 212157_at/SDC2, and 208949_s_at/LGALS3). In the overall chemotherapy cohort (non-responders n = 1100 vs responders n = 532), boxplot comparisons showed significantly higher expression of *FN1* and *VWF* in responders (*P <* 0.05), whereas *SDC2* was significantly higher in non-responders (*P <* 0.05) (Fig 9). Consistent with these expression differences, ROC analysis demonstrated statistically significant but modest discrimination for chemotherapy response with *FN1* (AUC = 0.573; *P <* 0.001), *VWF* (AUC = 0.568; *P <* 0.001), and *SDC2* (AUC = 0.597; *P <* 0.001) (Fig 10). In the overall anti-HER2 cohort (non-responders n = 122 vs responders n = 95), boxplots indicated that *FN1*, *VWF*, and *LGALS3* were significantly higher in non-responders (*P <* 0.05), supporting an association with resistance to anti-HER2 therapy (Fig 9). ROC curves similarly showed statistically significant but modest discrimination of anti-HER2 response status with *FN1* (AUC = 0.578; *P <* 0.05), *VWF* (AUC = 0.589; *P <* 0.05), and *LGALS3* (AUC = 0.596; *P <* 0.01) (Fig 10).

We further evaluated the association of these markers’ expression with individual treatment regimens. *FN1* was significantly associated with response to Taxane (AUC = 0.546; *P <* 0.01), Anthracycline (AUC = 0.574; *P <* 0.001), and FAC (AUC = 0.591; *P <* 0.05), but showed an inverse association in FEC, where higher expression was observed in non-responders (AUC = 0.611; *P <* 0.001). *VWF* was significantly associated with response to Anthracycline (AUC = 0.570; *P <* 0.001) and FAC (AUC = 0.607; *P <* 0.01), while it was higher in non-responders receiving FEC (AUC = 0.629; *P <* 0.001). *SDC2* showed consistent associations with resistance across Taxane (AUC = 0.581; *P <* 0.001), Anthracycline (AUC = 0.597; *P <* 0.001), and Ixabepilone (AUC = 0.608; *P <* 0.05). In Trastuzumab-treated patients, *FN1*, *VWF*, and *LGALS3* showed statistically significant but modest discrimination between responders and non-responders (*FN1* AUC = 0.573; *P <* 0.05, *VWF* AUC = 0.573; *P <* 0.05, *LGALS3* AUC = 0.572; *P <* 0.05) (Figs 9 **and** 10). Overall, these findings indicate that *FN1*, *VWF*, *SDC2*, and *LGALS3* are significantly associated with therapy-response patterns in public breast cancer transcriptomic cohorts, while the observed ROC performance remains modest, supporting their role as response-associated candidate markers requiring independent validation.

### 3.11. Identification of drug-gene interactions for FN1, VWF, SDC2, and Gal-3

To explore therapeutic opportunities, DGIdb https://dgidb.org; accessed February 1st, 2025) was queried to identify approved and investigational compounds targeting FN1, VWF, SDC2, and Gal-3. Approved drugs are included in Table 2, while investigational agents are summarized in S5 Table. Among the four targets, VWF was associated with the greatest number of approved drugs, reflecting its central role in coagulation and vascular-related therapies. These included Caplacizumab, Factor VIII, Mitomycin, and Warfarin. For FN1, two approved agents—Dacarbazine and Ocriplasmin were identified, though neither is currently indicated for breast cancer. In contrast, SDC2 and Gal-3 were linked primarily to investigational compounds, with no approved drugs currently targeting these molecules directly. These findings identify existing approved agents with theoretical relevance to these targets and emphasize the need for further investigation into target-specific drug development for SDC2 and Gal-3.

**Table 2:** Potential compounds targeting FN1, VWF, SDC2, and Gal-3.

Drug	DrugBank ID	Approval Status	Clinical Indication (Disease/Condition)	Target	Interaction score
Dacarbazine	DB00851	Approved (United States)	Metastatic melanoma; Hodgkin lymphoma	FN1	0.267711787
Ocriplasmin	DB08888	Approved (United States)	Symptomatic vitreomacular adhesion	FN1	0.211351411
Caplacizumab	DB06081	Approved (United States)	Acquired thrombotic thrombocytopenic purpura (aTTP)	VWF	7.830569784
Factor VIII	DB14473	Approved (United States)	Hemophilia A	VWF	0.870063309
Heparin	DB01109	Approved (United States)	Prevention and treatment of thromboembolic disorders	VWF	0.16313687
Warfarin	DB00682	Approved (United States)	Prevention and treatment of thrombosis and thromboembolism	VWF	0.158193329
Mitomycin	DB00305	Approved (United States)	Antineoplastic therapy, including gastric and pancreatic cancers	VWF	0.217515827
Thalidomide	DB01041	Approved (United States)	Multiple myeloma; erythema nodosum leprosum	VWF	0.141091347
Ribavirin	DB00811	Approved (United States)	Chronic hepatitis C infection (in combination regimens)	VWF	0.137378417
Pentoxifylline	DB00806	Approved (United States)	Intermittent claudication associated with chronic occlusive arterial disease	VWF	0.14915371
Streptozocin	DB00428	Approved (United States)	Pancreatic neuroendocrine tumors/ islet-cell carcinoma	VWF	0.170229778
Phenylephrine	DB00388	Approved (United States)	Clinically important hypotension; vasoconstrictor	VWF	0.326273741
Vitamin B6 (Pyridoxine)	DB00165	Approved (United States)	Vitamin B6 deficiency; prevention of isoniazid-induced neuropathy	VWF	0.870063309
Methylene Blue	DB09241	Approved (United States)	Methemoglobinemia	VWF	0.141091347

## 4. Discussion

In this study, we demonstrated using nanoLC-MS/MS the profile the plasma small-EV proteomic signature of obese, chemotherapy-naïve breast cancer patients, and identified FN1 and VWF as cargo components. Subsequent bioinformatics and STRING network analyses revealed their interacting partners SDC2 and Gal-3 to be enriched in small EVs and MV-enriched EVs, respectively. Whereas FN1, VWF, and SDC2 levels were significantly higher in small-EVs of plasma of breast cancer patients with either advanced stage and/or TNBC (Figs 4 and 5), Gal-3 was less enriched in small-EVs, relative to those of control plasma. Interestingly, Gal-3 was significantly increased in breast tumor tissues (vs normal tissues) and MV-associated EVs in plasma of breast cancer patients in staging-dependent manner (Fig 5). Further, TNBC-derived small-EVs induced EMT-related changes in MCF-7 cells and triggered inflammatory programs in BNL CL.2 normal liver cells (Fig 6). Despite modest therapy-response discrimination in the ROC-plotter database (Figs 9 and 10), these findings suggest that FN1, VWF, SDC2, and Gal-3 may emerge as biologically relevant markers for disease progression monitoring and to stratify patients who would benefit from treatment decisions.

Small-EVs mediate intercellular communication and PMN formation by delivering cargo that modulates inflammation, vascular permeability, and ECM remodeling [13,44–46]. In this context, the enrichment of FN1, VWF, and SDC2 in plasma small-EVs—alongside their interaction networks—supports their role in metastatic conditioning. FN1 is a critical matrisome component involved in adhesion and dissemination [47] and frequently enriched in cancer-derived EVs [48]. Notably, surface FN1 enhances EV biogenesis, uptake, and its accumulation at pre-metastatic sites [49]. These observations align with our finding that FN1 is highly enriched in patient-derived small-EVs and particularly higher in TNBC subtype (Fig 4), as well as it may support its outperformance compared with the soluble plasma FN1 in breast cancer detection [50].

Our data identify SDC2 as a key EV-associated component in breast cancer progression (Fig 5). SDC2, a heparan sulfate proteoglycan, regulates ECM organization, focal adhesion signaling, and angiogenesis [36]. Elevated SDC2 is linked to vascular invasion and metastasis across tumor types [36]; in breast cancer, it specifically modulates stromal TGF-β signaling and EMT programs [51]. Notably, the enrichment of SDC2 in plasma small-EVs—particularly in advanced-stage disease and TNBC—along with FN1, strengthens the biological coherence of an ECM-remodeling signature (Figs 4 and 5). This is further supported by our *in-silico* analyses, where HPA single-cell RNA showed FN1 and SDC2 localized to stromal/fibroblast compartments, while UALCAN/CPTAC confirmed their increased tumor-associated protein abundance (Fig 7). To our knowledge, this is the first report that showed plasma-derived small-EVs from breast cancer patients contained SDC2. Of note, these results extend our previous observation that MV-enriched EVs of breast cancer patients with lymph node metastasis contained high FN1 and SDC2 [19], reinforcing FN1 and SDC2 as robust EV-associated markers for breast cancer progression and metastatic dissemination.

Beyond its classical role in coagulation [52], VWF contributes to vascular remodeling, endothelial activation, and metastatic dissemination [32]. In our cohort, VWF was strongly enriched in plasma small-EVs, especially in breast cancer with stage III (Fig 4), supporting its relevance as a circulating marker of cancer progression and aggressiveness. This aligns with studies reporting that VWF is confined in EVs in gastric cancer, NSCLC, melanoma, and glioblastoma [53–56]. Notably, our public-dataset analyses showed lower bulk-tumor VWF expression, whereas HPA single-cell RNA localized VWF predominantly to endothelial cells and IHC showed vascular-restricted staining (Fig 7). This may indicate that circulating EV-associated VWF reflects vascular or endothelial contributions to promote angiogenesis and metastasis [57]. Collectively, VWF-enriched small-EVs may serve as liquid-biopsy readouts of tumor–vascular activation in breast cancer.

Another important component of plasma small-EV cargo is Gal-3, a multifunctional lectin implicated in tumor progression, immune modulation, and TNBC therapy resistance [58,59]. Surprisingly, although it was detected in control plasma small-EVs but markedly reduced in breast-cancer small-EVs, Gal-3 was highly expressed in breast tumor tissues and contained in their MVs (in staging-dependent manner) (Figs 5 and 7). Its presence in normal small-EVs is biologically plausible as Gal-3 can be recruited into intraluminal vesicles via Tsg101 [60]. However, because Gal-3 lacks a classical signal peptide [39], it can also be secreted through unconventional pathways—including membrane blebbing and vesicular shedding—consistent with release in MV-like compartments [40]. These findings support a model where reduced Gal-3 in breast-cancer small-EVs reflects redistribution across extracellular compartments, being retained in tumor tissue or redirected toward MVs and soluble pools rather than reduced overall secretion [61]. This is supported by HPA single-cell RNA analysis, which localized *LGALS3* to immune/myeloid compartments, indicating that tissue-associated Gal-3 arises from both tumor and microenvironmental sources (Fig 7). Functionally, this redistribution remains compatible with pro-metastatic activity; circulating Gal-3 enhances endothelium-derived EV-mediated tumor adhesion via ICAM-1 [62] and supports matrix remodeling [63]. Secreted Gal-3 may also bind exosomal Gal3 BP—identified in our proteomics—to promote immunosuppressive PBMC polarization [64]. It should also be considered that circulating Gal-3 may partly reflect adsorption to MV/large-EV surfaces rather than intraluminal [65]. Future studies should further define its trafficking dynamics and how this affects the disease progression.

Given the role of EVs in local and distant metastasis via regulation of intercellular communication [43], TNBC-derived small-EVs altered expression of EMT-related factors (increased *ZEB2* and FN1and decreased CDH1) in MCF-7 breast cancer cells as well as the inflammatory, matrix-remodeling programs in BNL CL.2 normal liver cells (Fig 6). ZEB2 is a transcription factor involved in the suppression of E-cadherin, which is consistent with the observed decrease in CDH1 expression [41]. This aligns with models where TNBC-derived vesicles trigger EMT, where FN1 promotes pro-migratory signaling [66]. Further, the increased *Il-6*, *Tnf-α*, and *Mmp-9* expression in BNL CL.2 cells suggests inflammatory PMN conditioning. This is supported by evidence that TNBC EVs promote hepatic niches via TNFα-linked remodeling and MMP9-associated macrophage recruitment [67], as well as breast-cancer exosomes induce IL-6 and inflammatory macrophage polarization through gp130/STAT3 signaling [68]. Collectively, these data confirm that tumor EVs prime distant tissues through inflammatory activation and FN1-rich niche formation [69]. Beyond being biomarker carriers, patient-derived small-EVs act as active mediators that shape recipient-cell behavior to facilitate metastatic progression.

Although translationally perspective of our discovery study supported by biological validation, some limitations do exist. First, ROC-Plotter analyses linked *FN1*, *VWF*, *SDC2*, and *LGALS3* to therapy response had modest performance and transcript expression-based (Figs 9 and 10). Second, this research is focused on obese, chemotherapy-naïve patients, nonetheless, given the prevalence of obesity and its link to breast cancer in Egypt [6,7] and our treatment-naïve cohort remains highly relevant, minimizing therapy-related confounding. Additionally, while direct tracking of EV uptake was beyond this study’s scope, future labeling strategies are necessary to confirm internalization and mechanistic clues underlying the observed elevated FN1 in MCF7 cells upon incubation with TNBC-derived small EVs, whether it is transcription/translation/epigenetic mechanism (s) and/or direct delivery of FN1 content of small-EVs into recipient cells.

## 5. Conclusion

This study identified a distinct plasma small-EVs proteomic signature in obese, chemotherapy-naïve breast cancer patients, characterized by enrichment of FN1, VWF, and SDC2 and reduced Gal-3 in small-EVs, being differentially contained in breast cancer with different stages and TNBC molecular subtype. Importantly, Gal-3 was preferentially enriched in MV-associated EVs rather than small-EVs, in tumor staging-dependent manner. We proved using an in vitro model the intercellular communication and preparation of premetastatic niche by EVs, whereby TNBC-derived small-EVs induced EMT-associated transcriptional changes in MCF-7 breast cancer cells and promoted inflammatory and matrix-remodeling-related gene expressions in normal liver cells. Future retrospective and prospective studies are needed to validate and expand our findings using a multiplex protein assay in a large cohort including non-obese breast cancer patients. Furthermore, the determination of appropriate cutoff values for markers identified in enriched small-EVs and MV-enriched EVs will be necessary to evaluate their diagnostic, prognostic, and predictive utility, as well as their potential to distinguish between breast cancer stages and molecular subtypes. Finally, validation of the identified EV markers with respect to their association with tumor or immune cells is essential to clarify the cellular origin of these EVs.

## Supporting information

S1 Data Raw_images. (PDF)

S1 Fig Characterization of plasma-derived small extracellular vesicles (small-EVs). High resolution transmission electron microscopy (HR-TEM) images (scale bars:200 nm).
(PDF)

S2 Fig Representative western blots of PROS1, SDC4, and SDC1 in plasma derived small EVs from normal controls and breast cancer patients across stages and molecular subtypes. The uncropped full-length blot is provided in Supplementary S1_raw_images file.
(PDF)

S3 Fig mRNA expression of Fibronectin 1 (FN1), von Willebrand factor (VWF), Syndecan-2 (SDC2), and LGALS3 (Galectin-3; Gal-3) in the Molecular Taxonomy of Breast Cancer International Consortium (METABRIC) dataset. (A–D) Boxplots show mRNA expression scores for FN1 (A), VWF (B), SDC2 (C), and LGALS3 (D) in METABRIC (cBioPortal; https://www.cbioportal.org/; accessed 1 January 2025) across normal (n = 146) and breast cancer (n = 1826) samples, molecular subtypes (Luminal A (LumA), n = 700; Luminal B (LumB), n = 475; basal-like, n = 209; human epidermal growth factor receptor 2–enriched (HER2- enriched), n = 224), clinical stages (stage I, n = 501; stage II, n = 825; stage III, n = 118; stage IV, n = 10), histological grades (grade 1, n = 169; grade 2, n = 771; grade 3, n = 952), lymph node status (lymph node–negative (LN-), n = 993; lymph node–positive (LN+), n = 911), chemotherapy status (without chemotherapy, n = 1568; with chemotherapy, n = 412), and hormonal therapy status (without hormonal therapy, n = 764; with hormonal therapy, n = 1216). One-way analysis of variance (ANOVA) was used for >2-group comparisons, and Student’s t-test for two-group comparisons as indicated; *P < 0.05, **P < 0.001, ***P < 0.001.
(PDF)

S4 Fig Protein expression of Fibronectin 1 (FN1), von Willebrand factor (VWF), Syndecan-2 (SDC2), and Galectin-3 (Gal-3) in breast cancer proteomic datasets. Boxplots show Z-values for FN1, VWF, SDC2, and Gal-3 across (left) sample type (normal breast, n = 18; primary tumor, n = 125), (middle) clinical stage (normal, n = 18; stage I, n = 4; stage II, n = 74; stage III, n = 32), and (right) molecular subtype (normal, n = 18; luminal, n = 64; human epidermal growth factor receptor 2–positive (HER2-positive), n = 10; triple-negative breast cancer (TNBC), n = 16). P values are shown as indicated. Data were retrieved from UALCAN (https://ualcan.path.uab.edu/; accessed 2 January 2026).
(PDF)

S1 Table Antibodies used in this study. (PDF)

S2 Table Primer sequences used in this study. (PDF)

S3 Table Comparative proteomic profile of EVs proteins across early and late stages: shared, unique, and common protein sets. (PDF)

S4 Table Clinicopathologic characteristics of breast cancer patient’s plasma derived MV enriched fraction and normal controls. (PDF)

S5 Table Investigational and Non-Approved Compounds Targeting FN1, VWF, SDC2, and Gal-3. (PDF)

S1_File Raw proteomics results for stage IA. (XLSX)

S2_File Raw proteomics results for stage IIA. (XLSX)

S3_File Raw proteomics results for stage IIB. (XLSX)

S4_File Raw proteomics results for stage IIIA and IIIC. (XLSX)

S5_File Raw proteomics results for stage IIIA. (XLSX)

S6_File Raw proteomics results for stage IIIA, IIIB, and IIIC. (XLSX)

## Abbreviations

ECM: Extracellular matrix
ACD: Acid Citrate Dextrose
AUC: The area under curve
BCS: Breast conservative surgery
BMI: Body mass index
DGIdb: The Drug Gene Interaction Database
ECL: Enhanced chemiluminescence
EMT: Epithelial-to-mesenchymal transition
ER: Estrogen-receptor
EVs: Extracellular vesicles
FN1: Fibronectin
HER2: Human epidermal growth factor-2
HR-TEM: High-resolution transmission electron microscopy
IHC: Immunohistochemical
LGALS3: Galectin 3
MRM: Modified radical mastectomy
PBS: Phosphate-buffered saline
PEG: Polyethylene glycol
PMN: Pre-metastatic niche
PPI: Protein–protein interaction
PR: Progesterone-receptor
PROS1: Protein S
ROC: Receiving operating characterstics
SDC2: Syndecan 2
TME: Tumor microenvirnment
TNBC: Triple-negative breast cancer
VWF: Von Willebrand Factor
