# Supplementary material for: Plasma small-extracellular vesicles’ proteomic signature in neoadjuvant chemotherapy–naïve breast cancer patients
Source: PLoS One. 2026 May 5;21(5):e0348500. doi: 10.1371/journal.pone.0348500 (PMC13143105; doi:10.1371/journal.pone.0348500)
Supplement: S1 Data — (PDF) [file pone.0348500.s001.pdf]

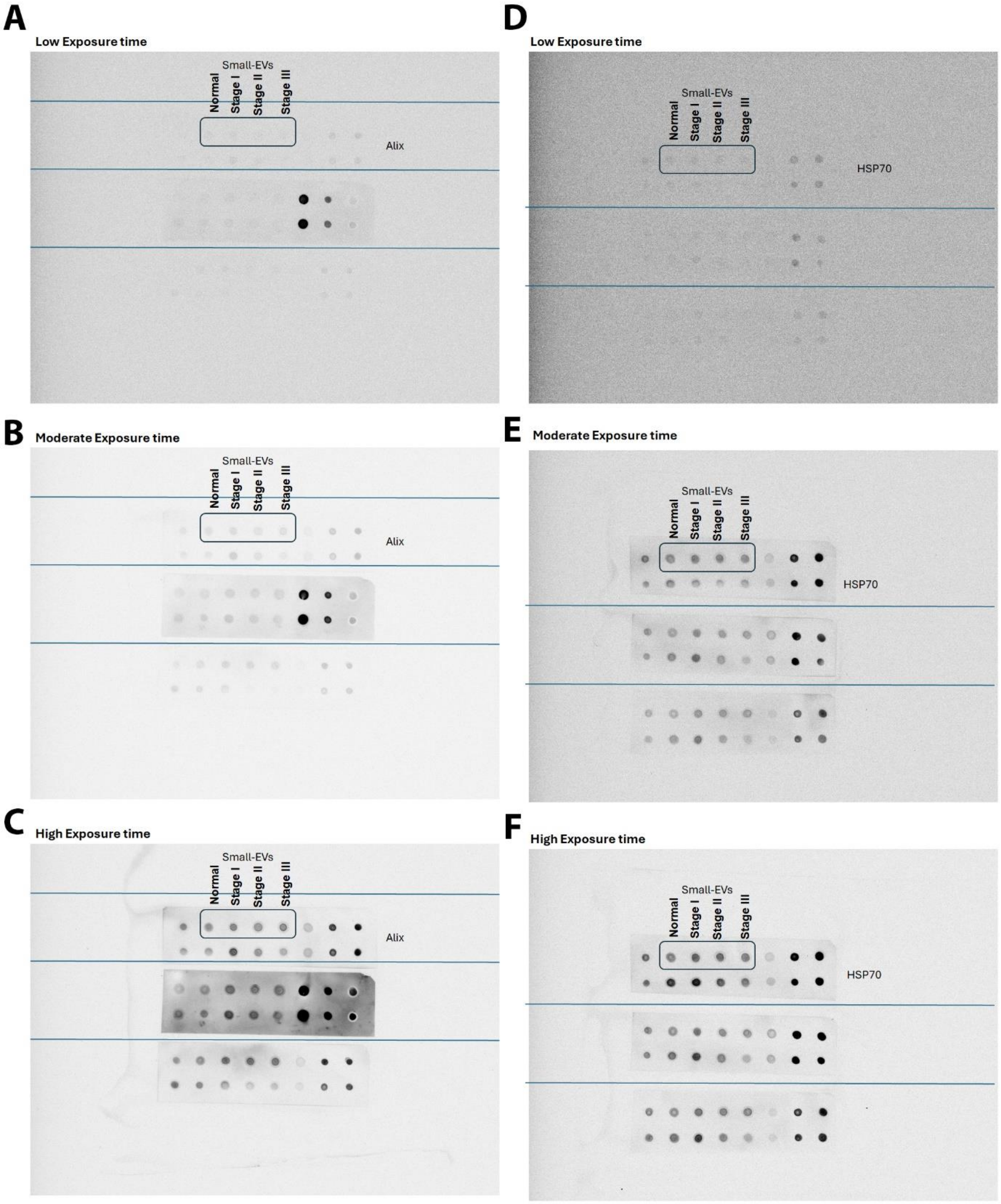

Raw data for Fig 1C (ALIX and HSP70). Original uncropped full-length unprocessed versions with different exposure time. (A-B) ALIX original images (A) Low exposure time. (B) moderate exposure time. (C) High exposure time. (D_F) HSP70 original images (D) Low exposure time. (E) moderate exposure time. (F) High exposure time.

A

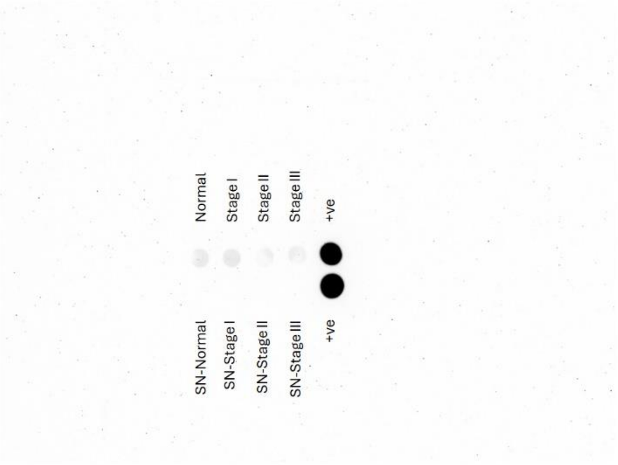

B

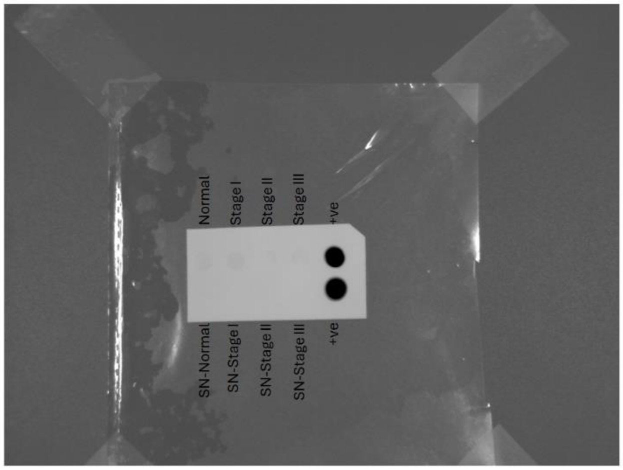

Raw data for Fig 1D (Calnexin). Original uncropped full-length unprocessed versions. (A) Moderate exposure time. (B) Merged membrane and moderate exposure time

A

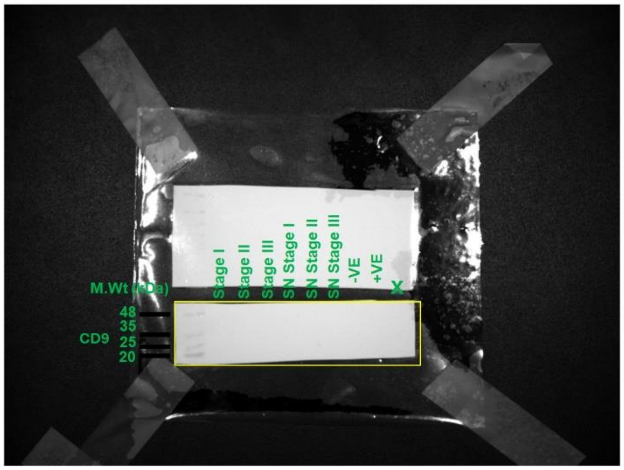

B

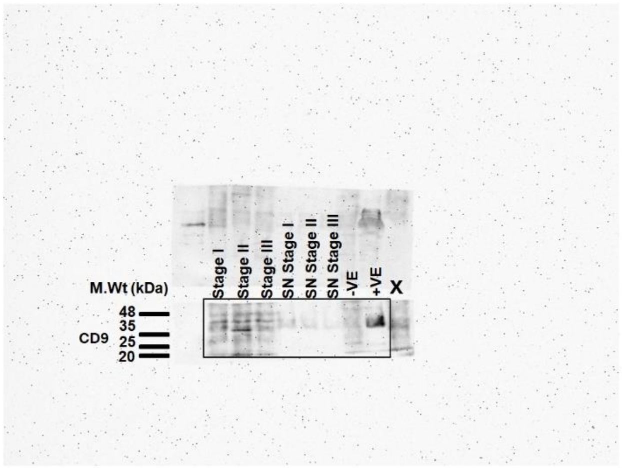

Raw data for Fig 1E (CD9). Original uncropped full-length unprocessed versions. (A) Original membrane with marker. (B) Moderate exposure time.

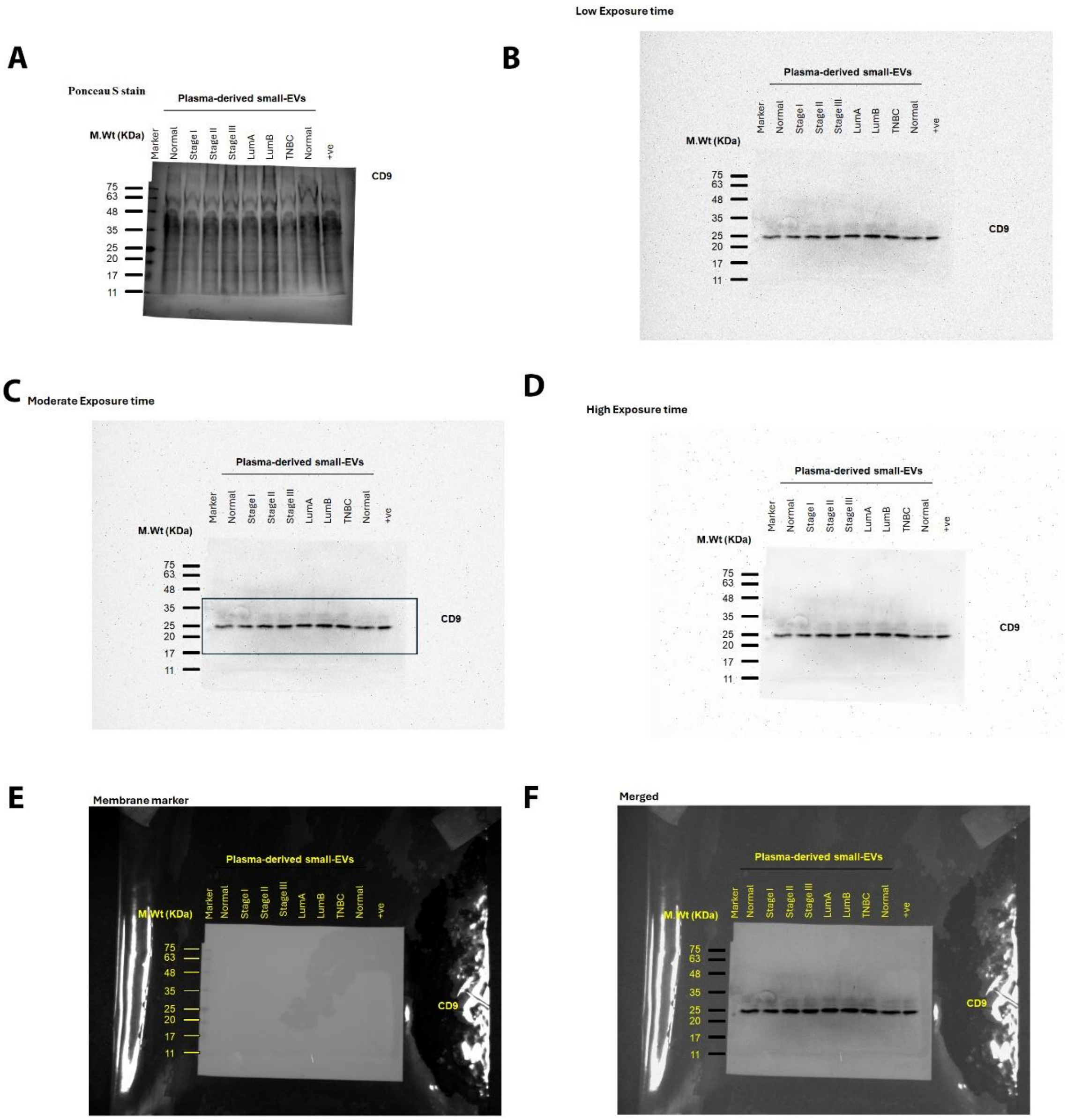

Raw data for Fig 1F (CD9). Original uncropped full-length unprocessed versions with different exposure time. (A) Ponceau S stain. (B) Low exposure time. (C) Moderate exposure time. (D) High exposure time. (E) Original membrane with marker. (F) Merged membrane with bands.

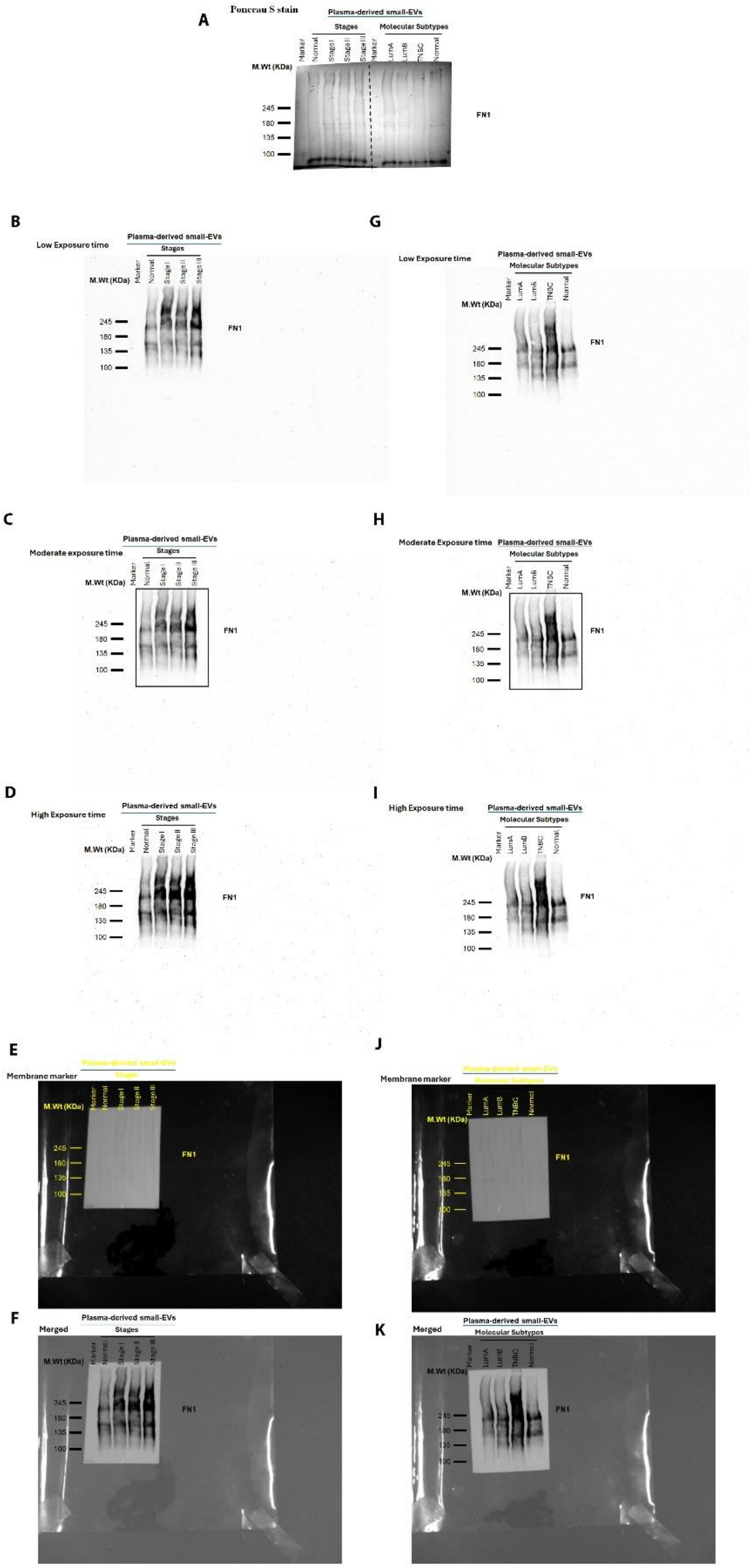

Raw data for Fig 4A (FN1). Original uncropped full-length unprocessed versions with different exposure time. (A) Ponceau S stain. (B-F) original images for FN1 across stages (B) Low exposure time. (C) Moderate exposure time. (D) High exposure time. (E) Original membrane with marker. (F) Merged membrane with bands. (G-K) original images for FN1 across Molecular subtypes. (G) Low exposure time. (H) Moderate exposure time. (I) High exposure time. (J) Original membrane with marker. (K) Merged membrane with bands.

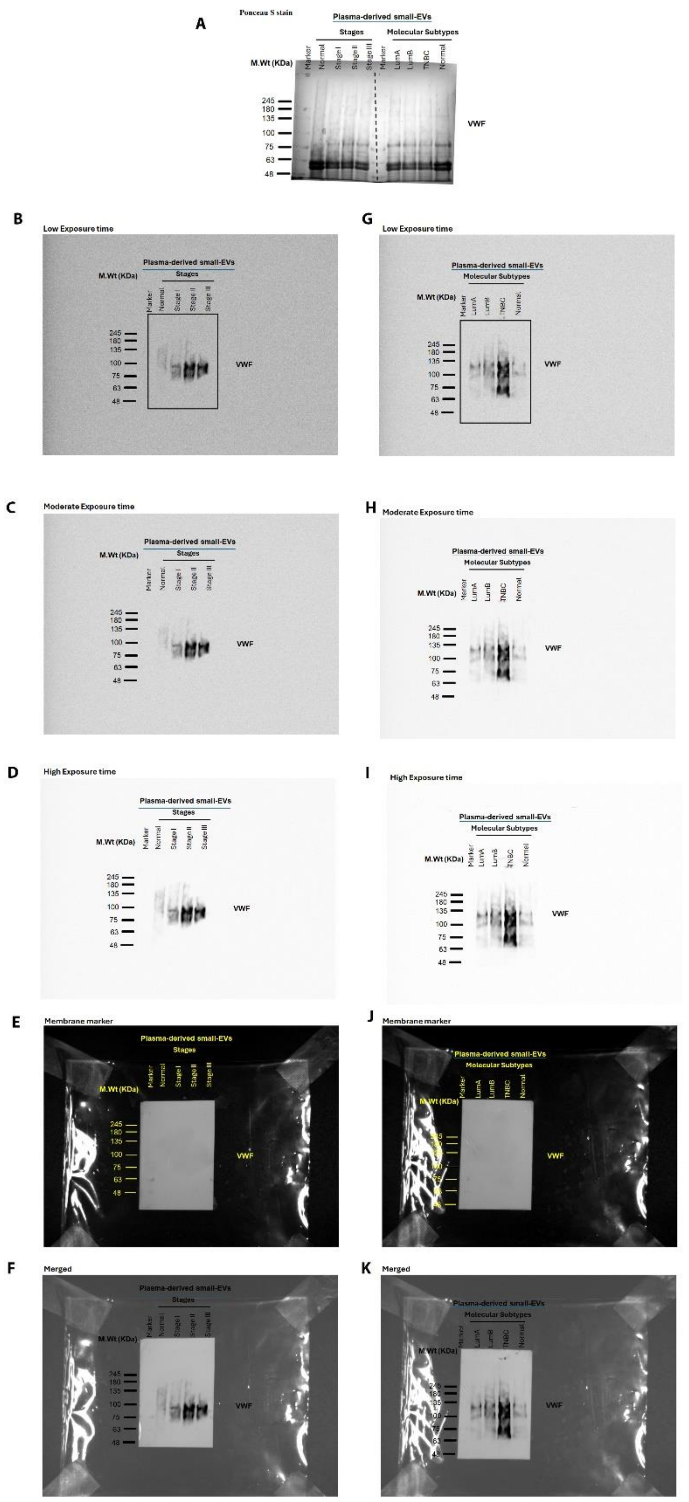

Raw data for Fig 4A (VWF). Original uncropped full-length unprocessed versions with different exposure time. (A) Ponceau S stain. (B-F) original images for VWF across stages (B) Low exposure time. (C) Moderate exposure time. (D) High exposure time. (E) Original membrane with marker. (F) Merged membrane with bands. (G-K) original images for VWF across Molecular subtypes. (G) Low exposure time. (H) Moderate exposure time. (I) High exposure time. (J) Original membrane with marker. (K) Merged membrane with bands.

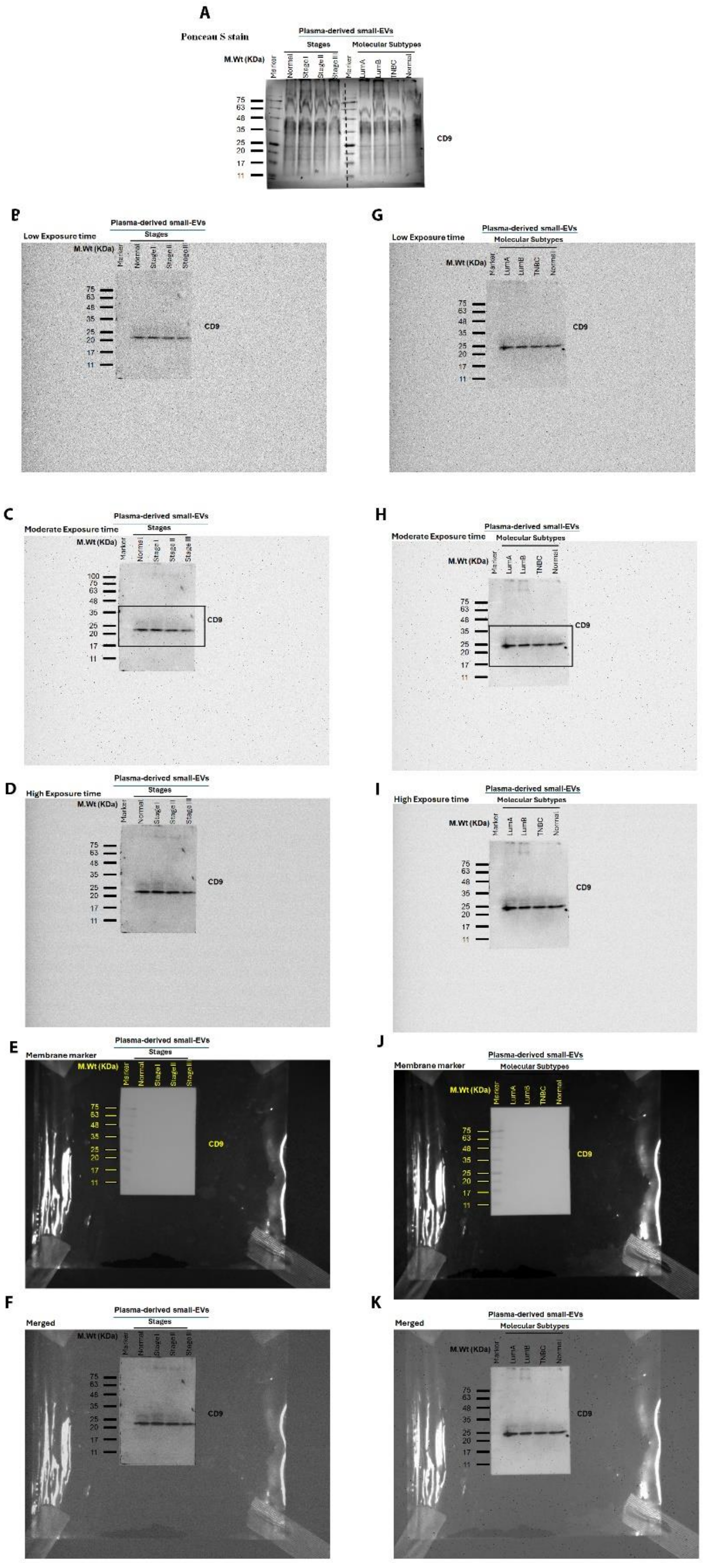

Raw data for Fig 4A (CD9). Original uncropped full-length unprocessed versions with different exposure time. (A) Ponceau S stain. (B-F) original images for CD9 across stages (B) Low exposure time. (C) Moderate exposure time. (D) High exposure time. (E) Original membrane with marker. (F) Merged membrane with bands. (G-K) original images for CD9 across Molecular subtypes. (G) Low exposure time. (H) Moderate exposure time. (I) High exposure time. (J) Original membrane with marker. (K) Merged membrane with bands.

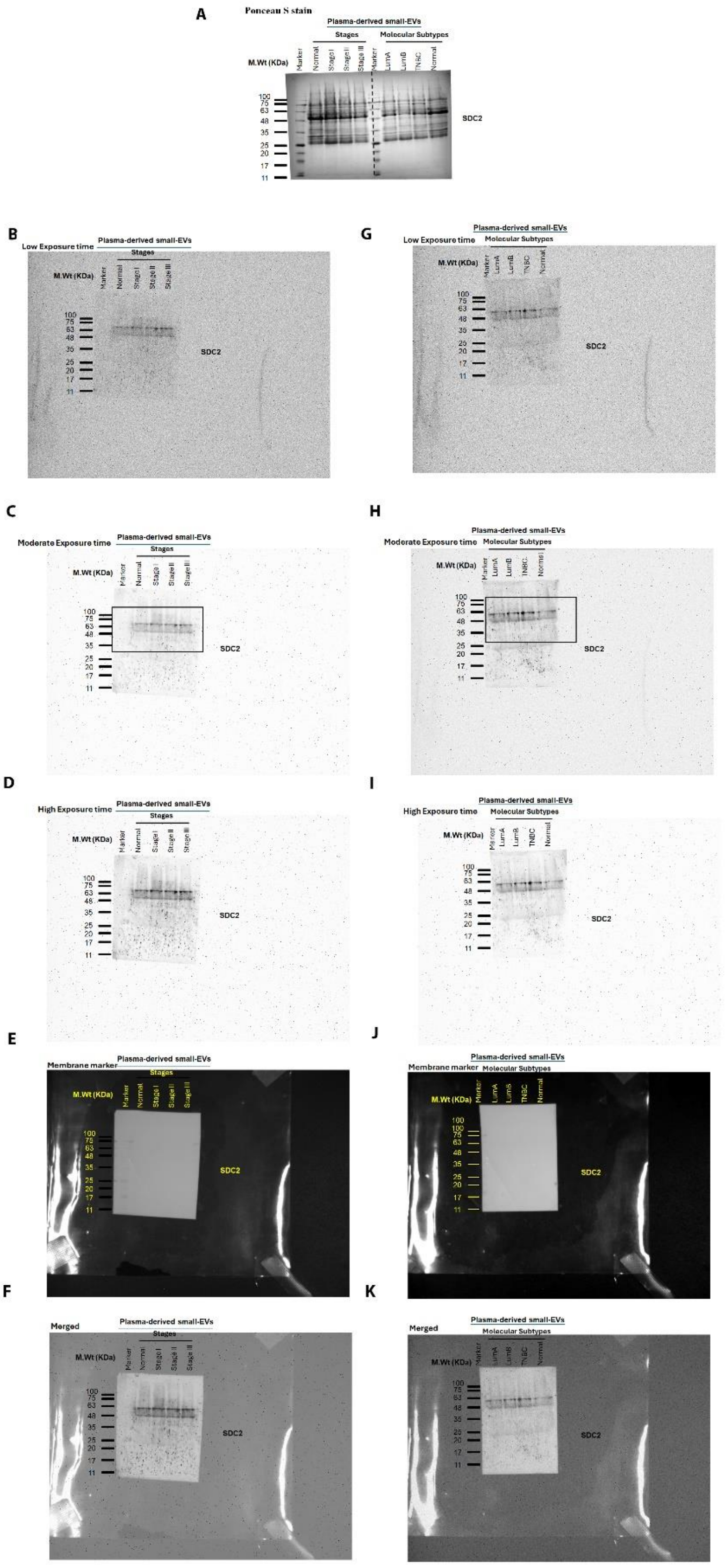

Raw data for Fig 5C (SDC2). Original uncropped full-length unprocessed versions with different exposure time. (A) Ponceau S stain. (B-F) original images for SDC2 across stages (B) Low exposure time. (C) Moderate exposure time. (D) High exposure time. (E) Original membrane with marker. (F) Merged membrane with bands. (G-K) original images for SDC2 across Molecular subtypes. (G) Low exposure time. (H) Moderate exposure time. (I) High exposure time. (J) Original membrane with marker. (K) Merged membrane with bands.

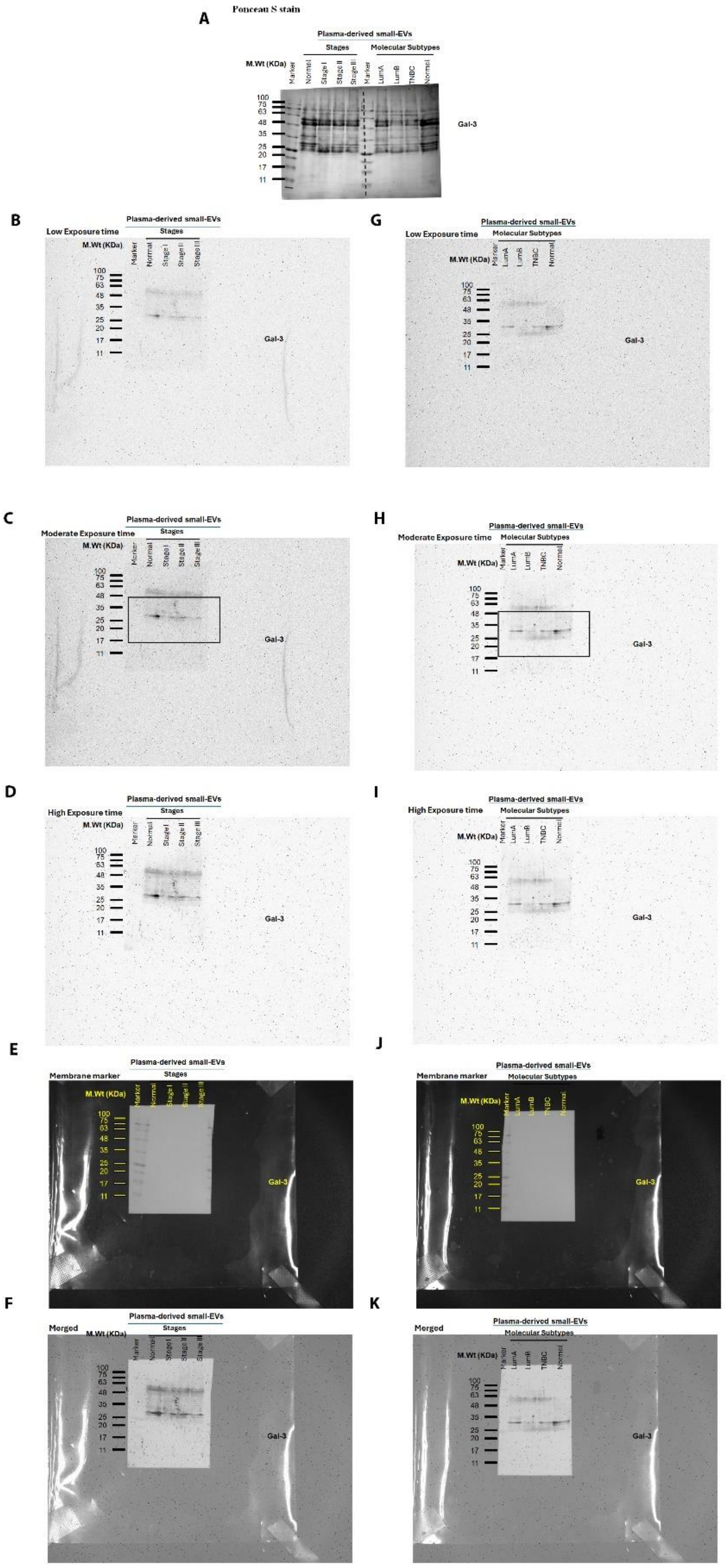

Raw data for Fig 5C (Gal-3). Original uncropped full-length unprocessed versions with different exposure time. (A) Ponceau S stain. (B-F) original images for Gal-3 across stages (B) Low exposure time. (C) Moderate exposure time. (D) High exposure time. (E) Original membrane with marker. (F) Merged membrane with bands. (G-K) original images for Gal-3 across Molecular subtypes. (G) Low exposure time. (H) Moderate exposure time. (I) High exposure time. (J) Original membrane with marker. (K) Merged membrane with bands.

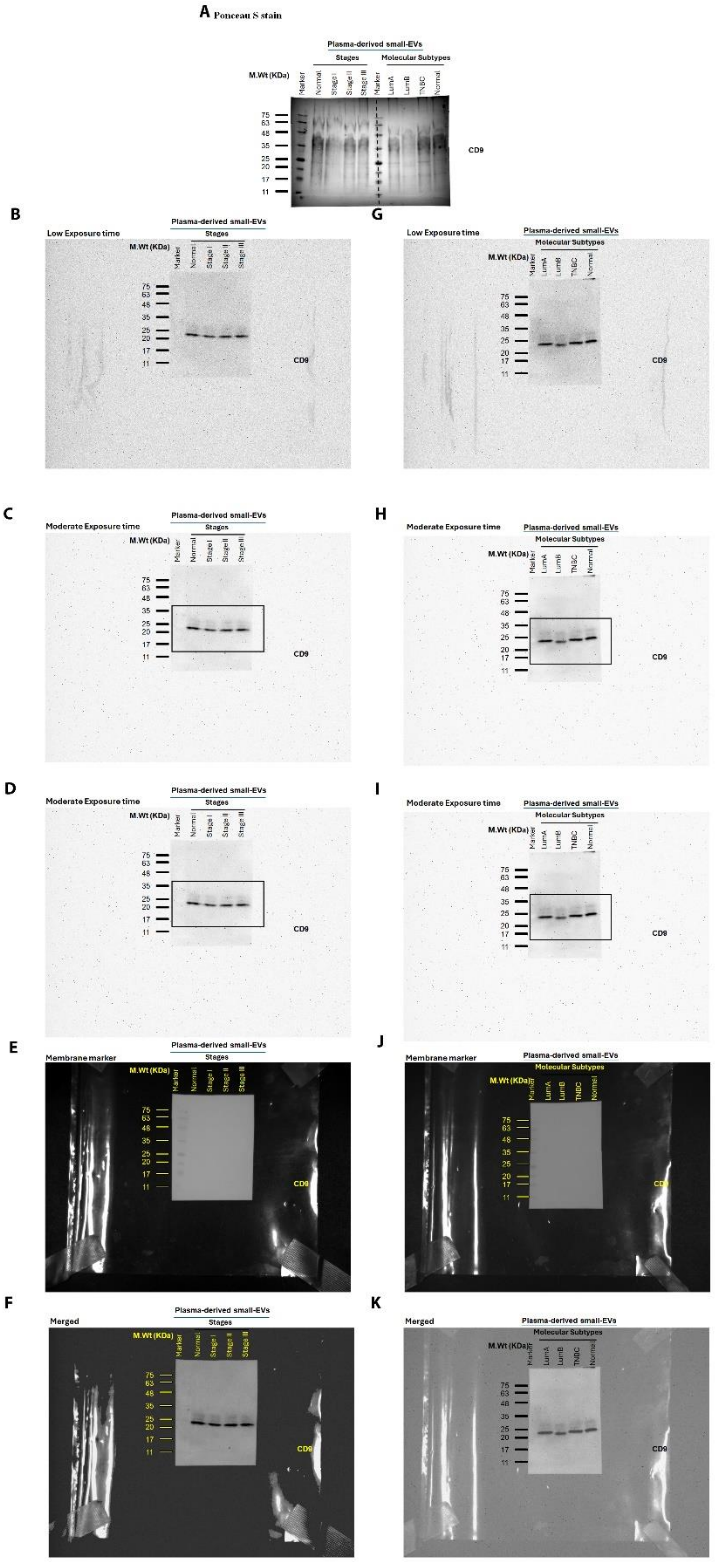

Raw data for Fig 5C (CD9). Original uncropped full-length unprocessed versions with different exposure time. (A) Ponceau S stain. (B-F) original images for CD9 across stages (B) Low exposure time. (C) Moderate exposure time. (D) High exposure time. (E) Original membrane with marker. (F) Merged membrane with bands. (G-K) original images for CD9 across Molecular subtypes. (G) Low exposure time. (H) Moderate exposure time. (I) High exposure time. (J) Original membrane with marker. (K) Merged membrane with bands.

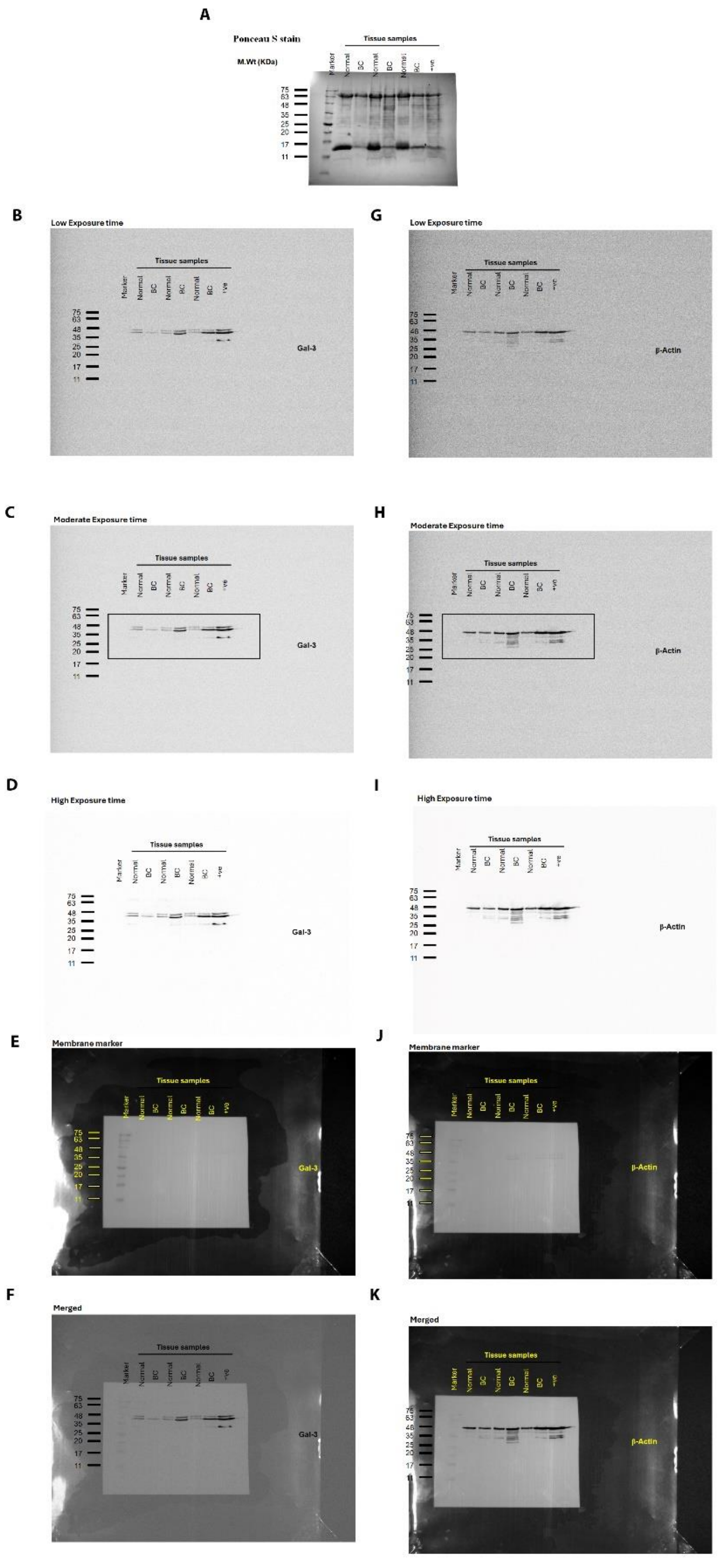

Raw data for Fig 5F (Gal-3 and β-Actin). Original uncropped full-length unprocessed versions with different exposure time. (A) Ponceau S stain. (B-F) original images for Gal-3 across samples (B) Low exposure time. (C) Moderate exposure time. (D) High exposure time. (E) Original membrane with marker. (F) Merged membrane with bands. (G-K) original images for β-Actin across samples. (G) Low exposure time. (H) Moderate exposure time. (I) High exposure time. (J) Original membrane with marker. (K) Merged membrane with bands.

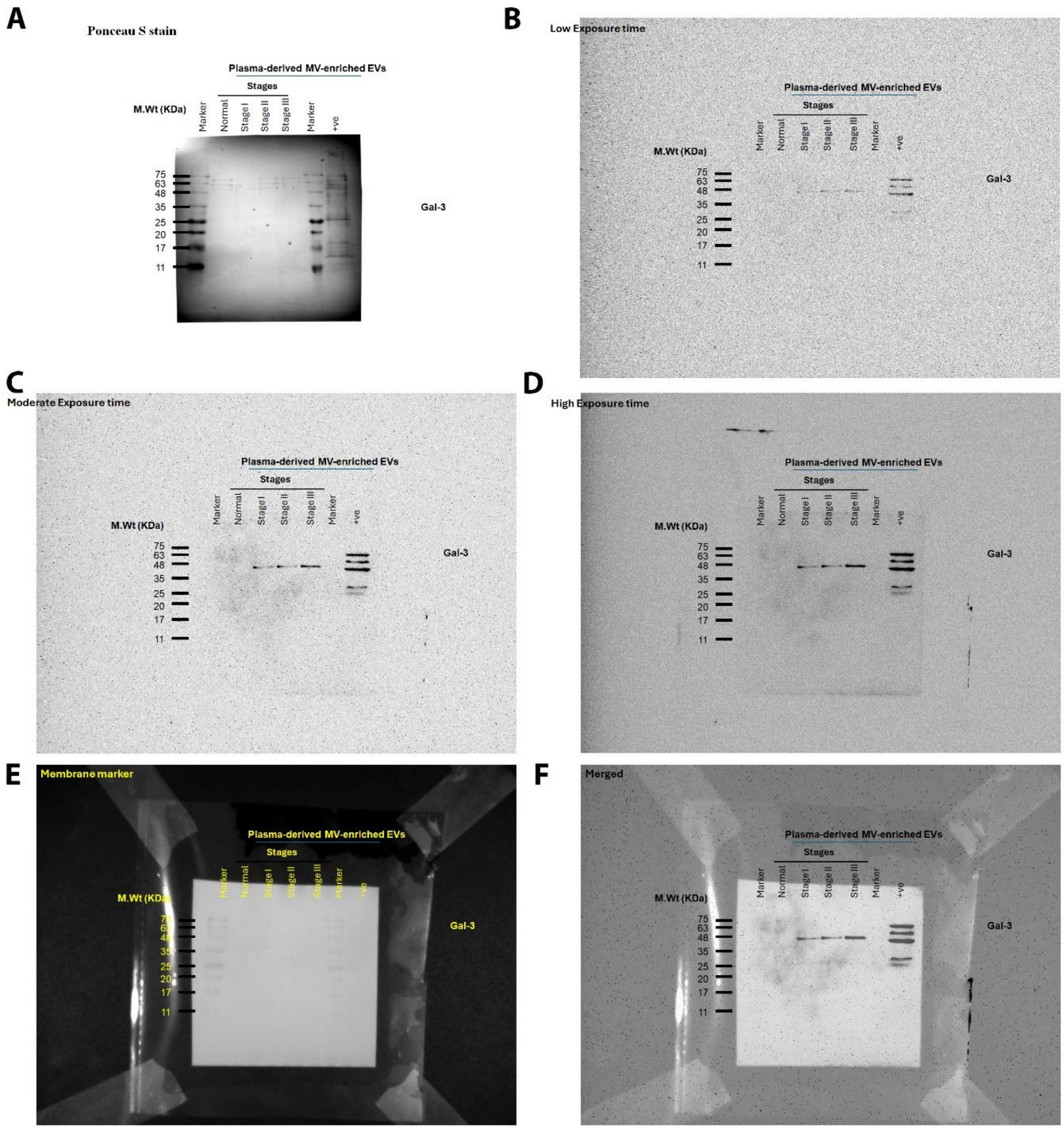

Raw data for Fig 5H (Gal-3). Original uncropped full-length unprocessed versions with different exposure time. (A) Ponceau S stain. (B) Low exposure time. (C) Moderate exposure time. (D) High exposure time. (E) Original membrane with marker. (F) Merged membrane with bands.

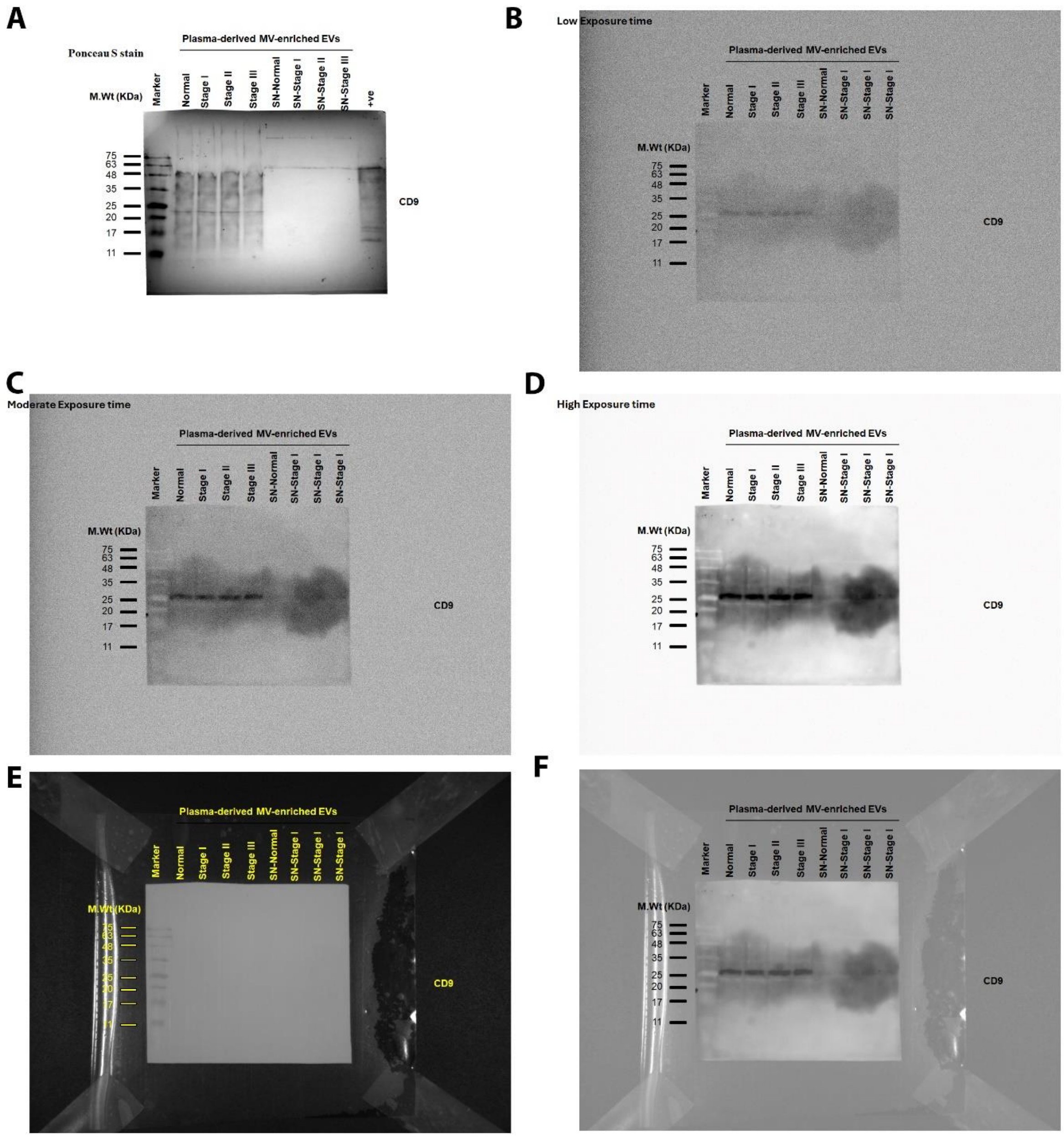

Raw data for Fig 5C (CD9). Original uncropped full-length unprocessed versions with different exposure time. (A) Ponceau S stain. (B) Low exposure time. (C) Moderate exposure time. (D) High exposure time. (E) Original membrane with marker. (F) Merged membrane with bands.

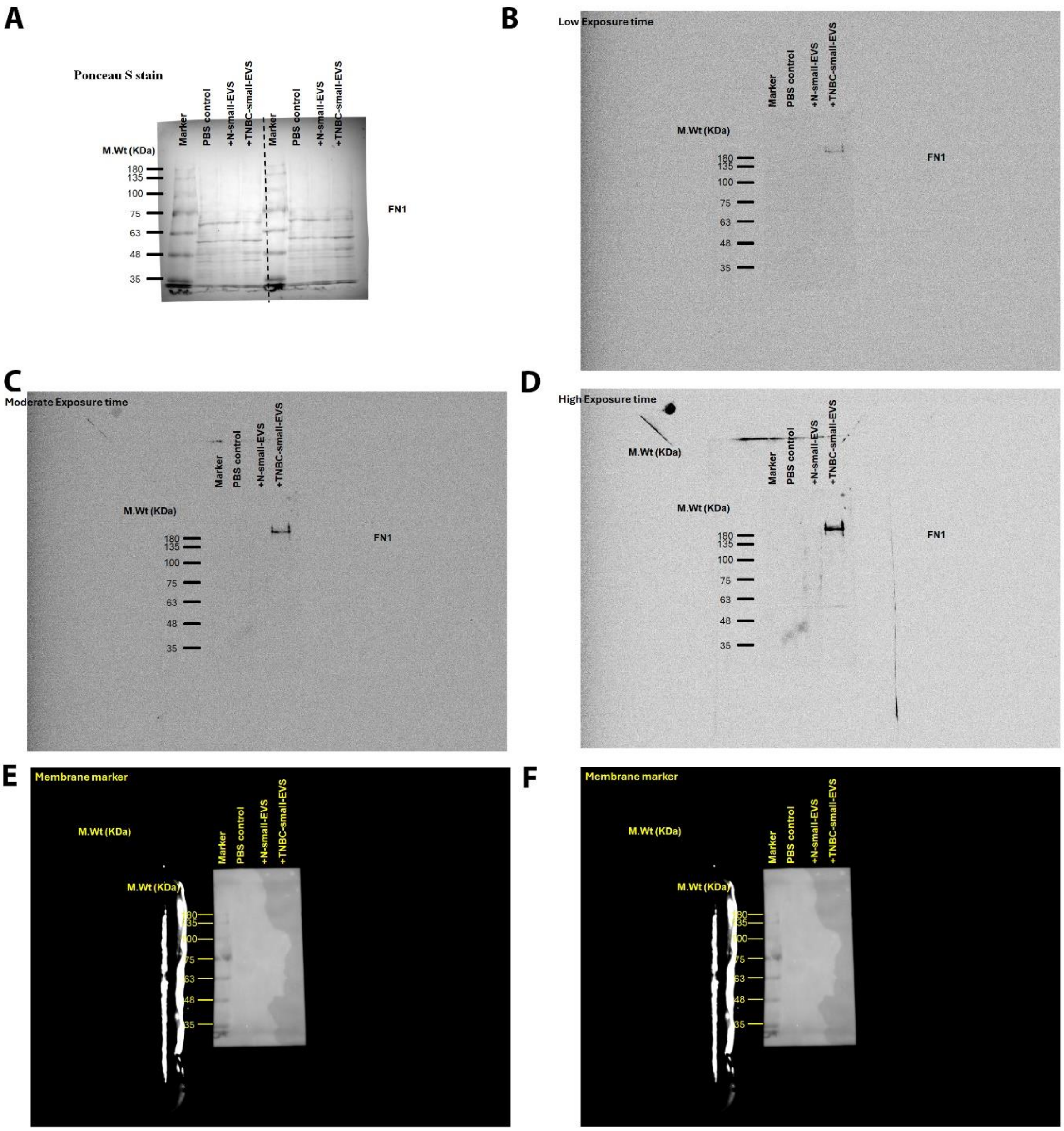

Raw data for Fig 5B (FN1). Original uncropped full-length unprocessed versions with different exposure time. (A) Ponceau S stain. (B) Low exposure time. (C) Moderate exposure time. (D) High exposure time. (E) Original membrane with marker. (F) Merged membrane with bands.

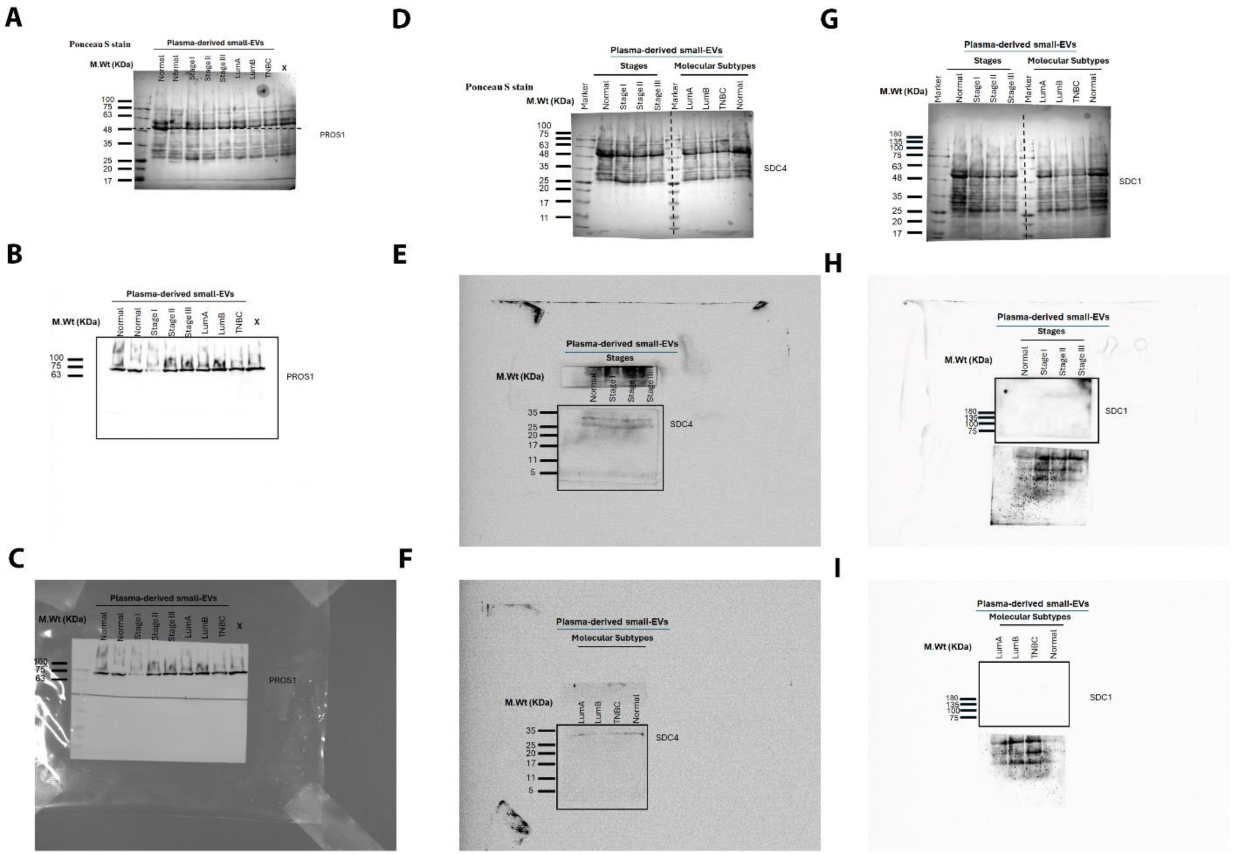

Raw data for supplementary Fig S1 (PROS1, SDC1, and SDC4). Original uncropped full-length unprocessed versions with different exposure time. (A-C) original images for PROS1. (D-F) original images for SDC4. (G-H) original images for SDC1.
