# Supplementary material for: Plasma small-extracellular vesicles’ proteomic signature in neoadjuvant chemotherapy–naïve breast cancer patients
Source: PLoS One. 2026 May 5;21(5):e0348500. doi: 10.1371/journal.pone.0348500 (PMC13143105; doi:10.1371/journal.pone.0348500)
Supplement: S1 Fig — High resolution transmission electron microscopy (HR-TEM) images (scale bars:200 nm). (PDF) [file pone.0348500.s002.pdf]

Supplementary S1 Fig.

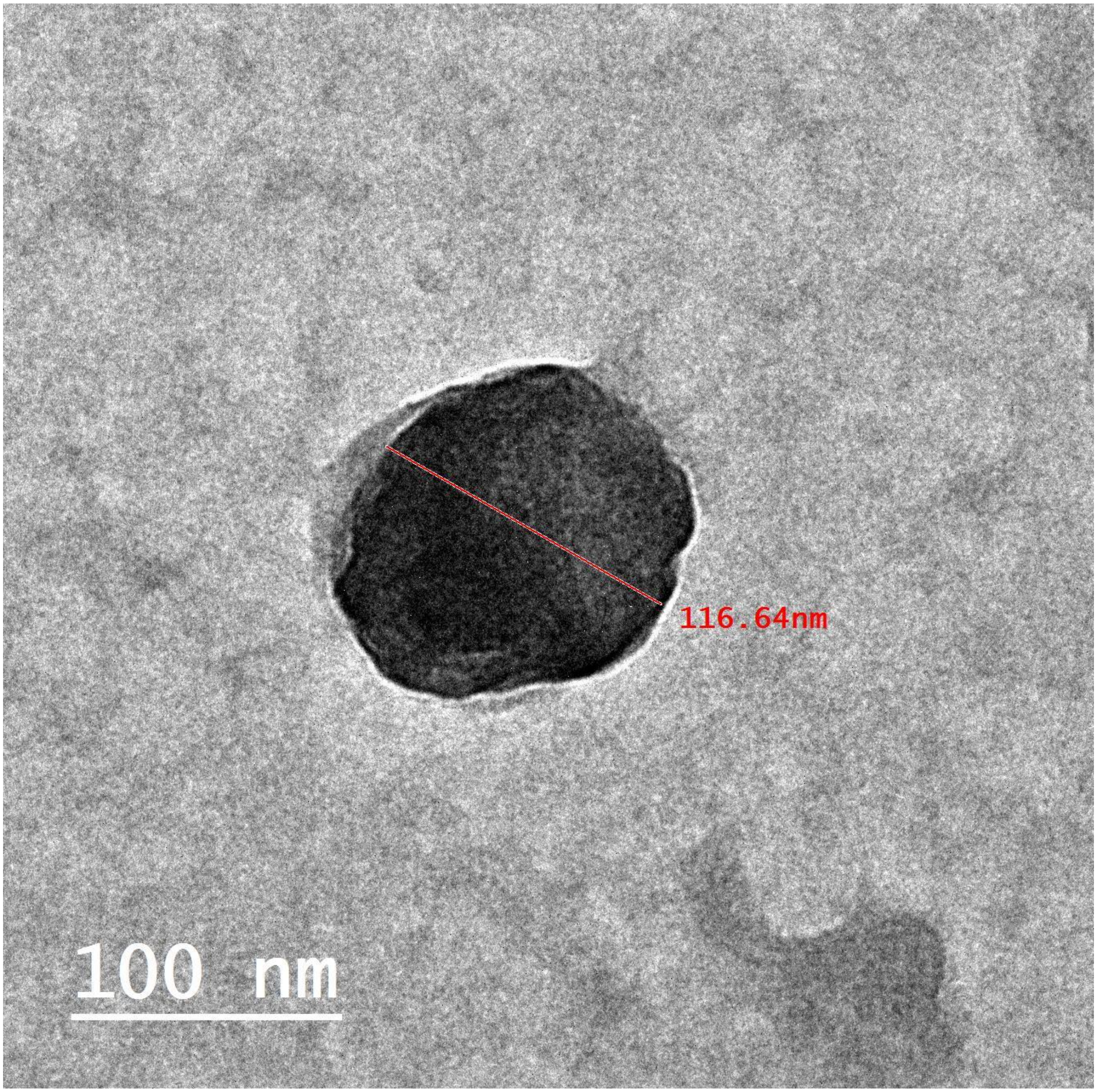

Supplementary. S1 Fig. Characterization of plasma-derived small extracellular vesicles (small-EVs). High-resolution transmission electron microscopy (HR-TEM) images (scale bars: 200 nm).
