# Supplementary material for: Plasma small-extracellular vesicles’ proteomic signature in neoadjuvant chemotherapy–naïve breast cancer patients
Source: PLoS One. 2026 May 5;21(5):e0348500. doi: 10.1371/journal.pone.0348500 (PMC13143105; doi:10.1371/journal.pone.0348500)
Supplement: S2 Fig — The uncropped full-length blot is provided in Supplementary S1_raw_images file. (PDF) [file pone.0348500.s003.pdf]

Supplementary S2 Fig.

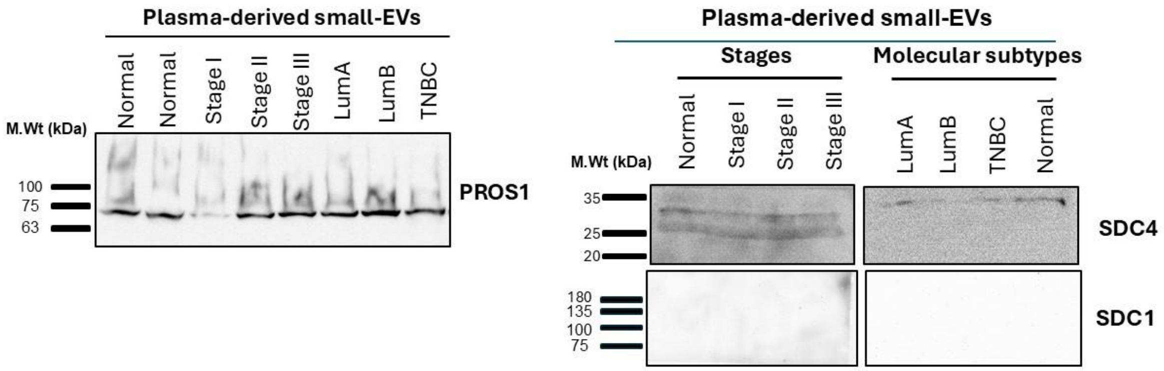

Supplementary. S2 Fig. Representative western blots of PROS1, SDC4, and SDC1 in plasma-derived small-EVs from normal controls and breast cancer patients across stages and molecular subtypes. The uncropped full-length blot is provided in Supplementary S1_raw_images file
