# Supplementary material for: Plasma small-extracellular vesicles’ proteomic signature in neoadjuvant chemotherapy–naïve breast cancer patients
Source: PLoS One. 2026 May 5;21(5):e0348500. doi: 10.1371/journal.pone.0348500 (PMC13143105; doi:10.1371/journal.pone.0348500)
Supplement: S3 Fig — (A–D) Boxplots show mRNA expression scores for FN1 (A), VWF (B), SDC2 (C), and LGALS3 (D) in METABRIC (cBioPortal; https://www.cbioportal.org/; accessed 1 January 2025) across normal (n = 146) and breast cancer (n = 1826) samples, molecular subtypes (Luminal A (LumA), n = 700; Luminal B (LumB), n = 475; basal-like, n = 209; human epidermal growth factor receptor 2–enriched (HER2- enriched), n = 224), clinical stages (stage I, n = 501; stage II, n = 825; stage III, n = 118; stage IV, n = 10), histological grades (grade 1, n = 169; grade 2, n = 771; grade 3, n = 952), lymph node status (lymph node–negative (LN-), n = 993; lymph node–positive (LN+), n = 911), chemotherapy status (without chemotherapy, n = 1568; with chemotherapy, n = 412), and hormonal therapy status (without hormonal therapy, n = 764; with hormonal therapy, n = 1216). One-way analysis of variance (ANOVA) was used for >2-group comparisons, and Student’s t-test for two-group comparisons as indicated; *P < 0.05, **P < 0.001, ***P < 0.001. (PDF) [file pone.0348500.s004.pdf]

Supplementary. S3 Fig.

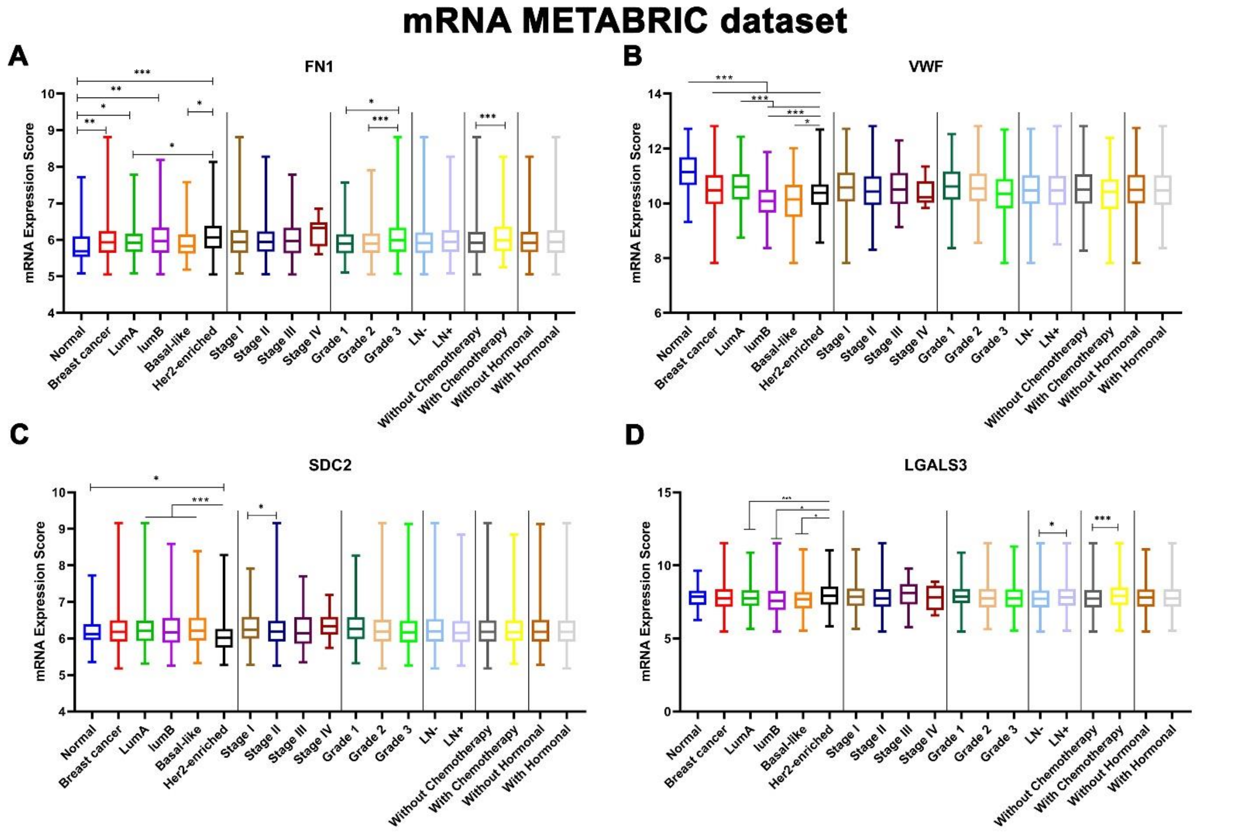

Supplementary. S3 Fig. mRNA expression of Fibronectin 1 (FN1), von Willebrand factor (VWF), Syndecan-2 (SDC2), and LGALS3 (Galectin-3; Gal-3) in the Molecular Taxonomy of Breast Cancer International Consortium (METABRIC) dataset. (A–D) Boxplots show mRNA expression scores for FN1 (A), VWF (B), SDC2 (C), and LGALS3 (D) in METABRIC (cBioPortal; https://www.cbioportal.org/; accessed 1 January 2025) across normal (n=146) and breast cancer (n=1826) samples, molecular subtypes (Luminal A (LumA), n=700; Luminal B (LumB), n=475; basal-like, n=209; human epidermal growth factor receptor 2–enriched (HER2-enriched), n=224), clinical stages (stage I, n=501; stage II, n=825; stage III, n=118; stage IV, n=10), histological grades (grade 1, n=169; grade 2, n=771; grade 3, n=952), lymph node status (lymph node–negative (LN−), n=993; lymph node–positive (LN+), n=911), chemotherapy status (without chemotherapy, n=1568; with chemotherapy, n=412), and hormonal therapy status (without hormonal therapy, n=764; with hormonal therapy, n=1216). One-way analysis of variance (ANOVA) was used for >2-group comparisons, and Student's t-test for two-group comparisons as indicated; *P < 0.05, **P < 0.001, ***P < 0.001.
