# Supplementary material for: Plasma small-extracellular vesicles’ proteomic signature in neoadjuvant chemotherapy–naïve breast cancer patients
Source: PLoS One. 2026 May 5;21(5):e0348500. doi: 10.1371/journal.pone.0348500 (PMC13143105; doi:10.1371/journal.pone.0348500)
Supplement: S4 Fig — Boxplots show Z-values for FN1, VWF, SDC2, and Gal-3 across (left) sample type (normal breast, n = 18; primary tumor, n = 125), (middle) clinical stage (normal, n = 18; stage I, n = 4; stage II, n = 74; stage III, n = 32), and (right) molecular subtype (normal, n = 18; luminal, n = 64; human epidermal growth factor receptor 2–positive (HER2-positive), n = 10; triple-negative breast cancer (TNBC), n = 16). P values are shown as indicated. Data were retrieved from UALCAN (https://ualcan.path.uab.edu/; accessed 2 January 2026). (PDF) [file pone.0348500.s005.pdf]

Supplementary. S4 Fig.

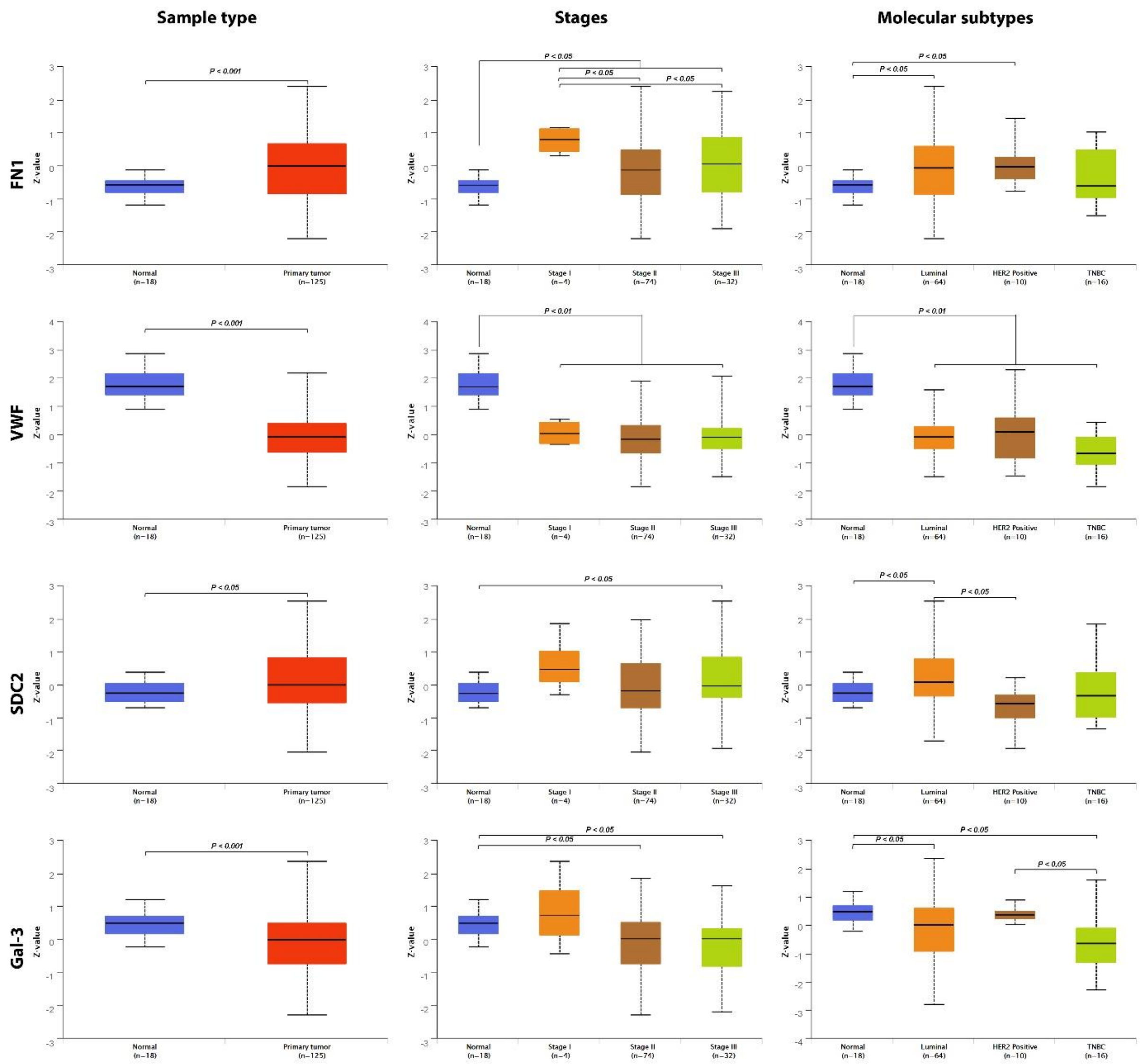

Supplementary. S4 Fig. Protein expression of Fibronectin 1 (FN1), von Willebrand factor (VWF), Syndecan-2 (SDC2), and Galectin-3 (Gal-3) in breast cancer proteomic datasets. Boxplots show Z-values for FN1, VWF, SDC2, and Gal-3 across (left) sample type (normal breast, n = 18; primary tumor, n = 125), (middle) clinical stage (normal, n = 18; stage I, n = 4; stage II, n = 74; stage III, n = 32), and (right) molecular subtype (normal, n = 18; luminal, n = 64; human epidermal growth factor receptor 2–positive (HER2-positive), n = 10; triple-negative breast cancer (TNBC), n = 16). P values are shown as indicated. Data were retrieved from UALCAN (https://ualcan.path.uab.edu/; accessed 2 January 2026).
