# Supplementary material for: Plasma small-extracellular vesicles’ proteomic signature in neoadjuvant chemotherapy–naïve breast cancer patients
Source: PLoS One. 2026 May 5;21(5):e0348500. doi: 10.1371/journal.pone.0348500 (PMC13143105; doi:10.1371/journal.pone.0348500)
Supplement: S1 Table — (PDF) [file pone.0348500.s006.pdf]

Supplementary Table S1

S1 Table. Antibodies used in this study.

Antibody	Working dilution	Manufacturer	City and Country
CD9	1:1000	Invitrogen (Ts9),	Carlsbad, USA
FN1	1:1000	Santa-Cruz (EP5): sc-8422	Dallas, TX, USA
VWF	1:1000	Santa-Cruz (G-11): sc-271409	Dallas, TX, USA
SDC1	1:1000	Santa-Cruz (DL-101): sc-12765	Dallas, TX, USA
SDC2	1:1000	Santa-Cruz (M-140): sc-15348	Dallas, TX, USA
SDC4	1:1000	Santa-Cruz (5G9): sc-12766	Dallas, TX, USA
PROS1	1:1000	Santa-Cruz (F-10): sc-271326	Dallas, TX, USA
Gal-3	1:1000	Santa-Cruz (B2C10): sc-32790	Dallas, TX, USA
HSP70	1:1000	Santa-Cruz (W27): sc-24	Dallas, TX, USA
CALNEXIN	1:1000	Santa-Cruz (H-70): sc-11397	Dallas, TX, USA
ALIX	1:1000	Santa-Cruz (1A12): sc-53540	Dallas, TX, USA
β-Actin	1:1000	Santa-Cruz (C4): sc-47778	Dallas, TX, USA
Goat Anti-Rabbit IgG, H & L chain specific peroxidase conjugate	1:2000	Merckmillipore (Cat: 401353)	Darmstadt, Germany
Goat Anti-mouse	1:2000	KPL (Cat: 04-18-06)	Gaithersburg, MD, USA
m-IgGκ BP-HRP	1:2000	Santa-Cruz sc-516102	Dallas, TX, USA
