# Supplementary material for: Plasma small-extracellular vesicles’ proteomic signature in neoadjuvant chemotherapy–naïve breast cancer patients
Source: PLoS One. 2026 May 5;21(5):e0348500. doi: 10.1371/journal.pone.0348500 (PMC13143105; doi:10.1371/journal.pone.0348500)
Supplement: S2 Table — (PDF) [file pone.0348500.s007.pdf]

S2 Table. Primer sequences used in this study.

Gene	Type	Sequences (5' - 3')
GAPDH	F	GTCTCCTCTGACTTCAACAGCG
	R	ACCACCCTGTTGCTGTAGCCAA
CDH1	F	GCCTCCTGAAAAGAGAGTGGAAG
	R	TGGCAGTGTCTCTCCAAATCCG
VIM	F	AGGCAAAGCAGGAGTCCACTGA
	R	ATCTGGCGTTCCAGGGACTCAT
ZEB2	F	AATGCACAGAGTGTGGCAAGGC
	R	CTGCTGATGTGCGAACTGTAGG
ACTB	F	CATTGCTGACAGGATGCAGAAGG
	R	TGCTGGAAGGTGGACAGTGAGG
Il-6	F	TACCACTTCACAAGTCGGAGGC
	R	CTGCAAGTGCATCATCGTTGTTC
Tnf-α	F	GGTGCCTATGTCTCAGCCTCTT
	R	GCCATAGAACTGATGAGAGGGAG
Mmp-2	F	CAAGGATGGACTCCTGGCACAT
	R	TACTCGCCATCAGCGTTCCCAT
Mmp-9	F	GCTGACTACGATAAGGACGGCA
	R	TAGTGGTGCAGGCAGAGTAGGA
