# Supplementary material for: Plasma small-extracellular vesicles’ proteomic signature in neoadjuvant chemotherapy–naïve breast cancer patients
Source: PLoS One. 2026 May 5;21(5):e0348500. doi: 10.1371/journal.pone.0348500 (PMC13143105; doi:10.1371/journal.pone.0348500)
Supplement: S3 Table — (PDF) [file pone.0348500.s008.pdf]

S3 Table. Comparative proteomic profile of EVs proteins across early and late stages: shared, unique, and common protein sets

Early stage shared with top 100 Evs proteins	Late stage shared with top 100 Evs proteins	Unique proteins in early stage	Unique protein in Late stage	Common between early stage and late stage	Shared 48 proteins in all stages
CLTC	A2M	ANLN	C8B	A2M	A2M
A2M	ALB	Bf	CMSS1	ADIC	AHSG
ALB	FN1	CAMP	FCN2	AHSG	ALB
FN1	GSN	CFHR5	IGKV1-9	ALB	APOA1
GSN	LGALS3BP	CSF2RB	IGLC7	APOA1	APOA2
LGALS3BP	ANXA1	CSPG4	MCOLN3	APOA2	APOA4
		EPS8L3	RAD21L	APOA4	APOB
		GDAP2	SETD1B	APOB	APOC1
		IGKV1-6	TAF1A	APOC1	APOC2
		LBP	TTF1	APOC3	APOC3
		LINC02694	V2-7	APOC4	APOC4
		NRAP	ZNF257	APOC4-APOC2	APOD
		PNPLA8	ZNFX1	APOD	APOE
		SHROOM3	ADH6	APOE	APOL1
		TEX13C	IGK@	APOL1	APOM
		TLE1	KRT1	APOM	C1QB
		V1-19	KRT10	C1QB	C1QC
		V1-3	LRRTM3	C1QC	C1R
		V5-6	MAP7D1	C1R	C1S
		BAAT	MTFMT	C1S	C3
		CCDC73	POP5	C4A	C4B
		ERC2	PRX	C4B_2	C4BPA
		FBXL20	SLC22A23	C4BPA	C4BPB
		FBXO15	SLIT1	C4BPB	C5
		hCG_2016179	SRRT	C5	C9
		HF	V2-17	C9	CD5L
		IGHV3-20	VL1	CD5L	CLU
		IGKV1-27	ANXA1	CFH	CTN
		IGKV2-29	C4B	CFI	F13A1
		IGKV3-11	DEFA3	CLU	F2
		IGKV3-15	FARP1	CRP	FCN3
		KRT2	IGHV2-5	DNM2	FGA
		KRTHA7	IWS1	F13A1	FGB
		LAMA2	KRT13	F2	FGG

		MAGED2	KRT9	FCN3	FN1
		MCAT	LSM4	FGA	GSN
		MTR	MASP1	FGG	HPX
		PKD2	SA	FN1	IGFALS
		RPL23	SPRR3	GSN	ITIH1
		SERPIND1	TRPV1	HEL-214	ITIH2
		SGA56M	V1-20	HEL-S-62p	ITIH4
		SGPP2		HEL-S-78p	JCHAIN
		SNC73		HMFT1766	LGALS3BP
		SORCS2		HP	LPA
		ZUBR1		HPR	PLG
		CLTC		HPX	PON1
		DENND3		IGFALS	PROS1
		EIF1AD		IGHM	VWF
		FBXO4		IGHV2-70	
		FLJ00385		IGHV3-30	
		HPSE2		IGHV3-53	
		IGHV4-30-4		IGHV3-66	
		MATN3		IGHV3-74	
		pp9964		IGHV5-51	
		SDNK1		IGKV1D-8	
		SFMBT2		IGKV2-24	
				ITIH1	
				ITIH2	
				ITIH4	
				JCHAIN	
				KNG1	
				LGALS3BP	
				LPA	
				LRRC32	
				PGLYRP2	
				PLG	
				PON1	
				PROS1	
				SAA2-SAA4	
				SHC1	
				V3-3	
				V3-4	

				V-kappa-1	
				VTN	
				VWF	
				ZIM3	
				C6	
				CFAP77	
				CFB	
				CPN1	
				F5	
				IgH	
				IGHV3-72	
				IGKV1D-39	
				KIDINS220	
				VH6DJ	
				CTCFL	
				HEL-S-163pA	
				HuVH8B	
				MASP2	
				SERPINF1	
				SMC1A	
				U34	
