# Supplementary material for: Plasma small-extracellular vesicles’ proteomic signature in neoadjuvant chemotherapy–naïve breast cancer patients
Source: PLoS One. 2026 May 5;21(5):e0348500. doi: 10.1371/journal.pone.0348500 (PMC13143105; doi:10.1371/journal.pone.0348500)
Supplement: S4 Table — (PDF) [file pone.0348500.s009.pdf]

S4 Table. Clinicopathologic characteristics of breast cancer patient's plasma derived MV-enriched fraction and normal controls.

Characteristic	Normal (n=36)	Stage I (n=15)	Stage II (n=26)	Stage III (n=10)	*P* value
**Age (years)**					
**Range**	36-79	34 - 76	36 - 79	48 - 79	*P* > 0.05
**Mean ± SEM**	54.31 ± 1.88	57.73 ± 3.523	55.35 ± 2.197	64.60 ± 2.952	
**< 50**	14 (38.78%)	5 (33.3%)	10 (38.5%)	1 (10%)	*P* > 0.05
**≥ 50**	22 (61.22%)	10 (66.7%)	16 (61.5%)	9 (90%)	
**Body mass index (BMI)**					
**Range**	30.66 – 48	30.76 - 47.67	30.41 - 48.83	30.18 - 45.81	*P* > 0.05
**Mean ± SEM**	37.01 ± 0.818	36.58 ± 1.433	37.40 ± 1.132	34.63 ± 1.430	
**Menopause status, n (%)**					
**Premenopausal**		4 (26.7%)	13 (50%)	1 (10%)	*P* > 0.05
**Postmenopausal**		8 (53.3%)	10 (38.5%)	7 (70%)	
**NA**		3 (20%)	3 (11.5%)	2 (20%)	
**Family history, n (%)**					
**Yes**		4 (26.7%)	4 (15.4%)	-	*P* > 0.05
**No**		11 (73.3%)	21 (80.8%)	10 (100%)	
**NA**		-	1 (3.8%)	-	
**Laterality, n (%)**					
**Bilateral**		1 (6.7%)	2 (7.7%)	-	*P* > 0.05
**Right**		3 (20%)	14 (53.8%)	4 (40%)	
**Left**		11 (73.3%)	10 (38.5%)	6 (60%)	
**Tumor size (cm), n (%)**					
**≤ 4**		14 (93.3%)	21 (80.8%)	8 (80%)	*P* > 0.05
**> 4**		1 (6.7%)	5 (19.2%)	2 (20%)	
**Tumor grade, n (%)**					
**Grade 1**		2 (13.3%)	1 (3.8%)	-	*P* > 0.05
**Grade 2**		11 (73.3%)	20 (76.9%)	8 (80%)	
**Grade 3**		2 (13.3%)	4 (15.4%)	2 (20%)	
**NA**		-	1 (3.8%)	-	
**ER, n (%)**					
**Negative**		1 (6.7%)	2 (7.7%)	-	*P* > 0.05
**Positive**		14 (93.3%)	24 (92.3%)	10 (100%)	

**PR, n (%)**					
**Negative**		-	2 (7.7%)	-	*P* > 0.05
**Positive**		15 (100%)	24 (92.3%)	10 (100%)	
**Her2, n (%)**					
**Negative**		13 (86.7%)	22 (84.6%)	7 (70%)	*P* > 0.05
**Equivocal (non-amplified)**		2 (13.3%)	4 (15.4%)	3 (30%)	
**Stages, n (%)**					
**IA**		14 (93.3%)	-	-	***P* < 0.05**
**IB**		1 (6.7%)	-		
**IIA**		-	16 (61.5%)	-	
**IIB**		-	10 (38.5%)	-	
**IIIA**		-	-	4 (40%)	
**IIIB**		-	-	2 (20%)	
**IIIC**		-	-	4 (40%)	
**Lymph node status, n (%)**					
**N0**		14 (93.3%)	14 (53.8%)	-	***P* < 0.05**
**N1**		1 (6.7%)	12 (46.2%)	2 (20%)	
**N2**		-	-	4 (40%)	
**N3**		-	-	4 (40%)	
**Tumor size, n (%)**					
**T1**		15 (100%)	4 (15.4%)	2 (20%)	***P* < 0.05**
**T2**		-	20 (76.9%)	5 (50%)	
**T3**		-	2 (7.7%)	1 (10%)	
**T4**		-	-	2 (20%)	
**Molecular subtypes**					
**Luminal A**		7 (46.7%)	11 (42.3%)	4 (40%)	*P* > 0.05
**Luminal B**		7 (46.7%)	12 (46.2%)	4 (40%)	
**Triple-negative**		1 (6.7%)	2 (7.7%)	-	
**NA**		-	1 (3.8%)	2 (20%)	
**Ki-67 % n (%)**					
**Low Ki-67 (< 20%)**		7 (46.7%)	13 (50%)	4 (40%)	*P* > 0.05
**High Ki-67 (≥ 20%)**		5 (33.3%)	9 (34.6%)	4 (40%)	
**NA**		3 (20%)	4 (15.4%)	2 (20%)	
