# Supplementary material for: Plasma small-extracellular vesicles’ proteomic signature in neoadjuvant chemotherapy–naïve breast cancer patients
Source: PLoS One. 2026 May 5;21(5):e0348500. doi: 10.1371/journal.pone.0348500 (PMC13143105; doi:10.1371/journal.pone.0348500)
Supplement: S5 Table — (PDF) [file pone.0348500.s010.pdf]

S5 Table. Investigational and Non-Approved Compounds Targeting FN1, VWF, SDC2, and Gal-3.

Drug	ID	Approval Status	Clinical Indication	Target
ONFEKAFUSP ALFA	NCIT:C94210	Not approved	Not available	FN1
COMPOUND 28 (PMID: 16460935)	PUBCHEM.SUBSTANCE:252166820	Not approved	Not available	FN1
L19TNFA	CHEMBL:CHEMBL2109589	Not approved	Not available	FN1
RADRETUMAB	CHEMBL:CHEMBL1743060	Not approved	Not available	FN1
DAROMUN	NCIT:C154627	Not approved	Not available	FN1
SUTIMLIMAB	PUBCHEM.SUBSTANCE:354702279	Not approved	Not available	FN1
MU-BC-1	CHEMBL:CHEMBL2109529	Not approved	Not available	FN1
L19IL2	CHEMBL:CHEMBL2109608	Not approved	Not available	FN1
AS-1409	CHEMBL:CHEMBL2109413	Not approved	Antineoplastic agent	FN1
L19SIP-131I	CHEMBL:CHEMBL2109412	Not approved	Not available	FN1
EGAPTIVON PEGOL	DB05202	Not approved	Antithrombotic	VWF
TNP-470	DB08633	Not approved	Not available	VWF
TYROSINE KINASE INHIBITOR	NCIT:C1967	Not approved	Not available	VWF
HEPARAN SULFATE	rxcui:2603494	Not approved	Not available	SDC2
OLITIGALTIN	CHEMBL:CHEMBL4297442	Not approved	Not available	Gal-3
DAVANAT	CHEMBL:CHEMBL4297960	Not approved	Not available	Gal-3
BELAPECTIN	CHEMBL:CHEMBL4297577	Not approved	Not available	Gal-3
LACTOSE, ANHYDROUS	CHEMBL:CHEMBL417016	Not approved	Not available	Gal-3
